# Dynamic and Sex-Specific Changes in Gonadotropin-Releasing Hormone Neuron Activity and Excitability in a Mouse Model of Temporal Lobe Epilepsy

**DOI:** 10.1523/ENEURO.0273-18.2018

**Published:** 2018-09-24

**Authors:** Jiang Li, Jordyn A. Robare, Liying Gao, M. Amin Ghane, Jodi A. Flaws, Mark E. Nelson, Catherine A. Christian

**Affiliations:** 1 Neuroscience Program, University of Illinois at Urbana-Champaign, Urbana, IL 61801; 2 Department of Molecular and Integrative Physiology, University of Illinois at Urbana-Champaign, Urbana, IL 61801; 3 Department of Comparative Biosciences, University of Illinois at Urbana-Champaign, Urbana, IL 61801; 4 Beckman Institute for Advanced Science and Technology, University of Illinois at Urbana-Champaign, Urbana, IL 61801

**Keywords:** excitability, GnRH, hormone, patch clamp electrophysiology, temporal lobe epilepsy

## Abstract

Reproductive endocrine disorders are prominent comorbidities of temporal lobe epilepsy (TLE) in both men and women. The neural mechanisms underlying these comorbidities remain unclear, but hypothalamic gonadotropin-releasing hormone (GnRH) neurons may be involved. Here, we report the first direct demonstrations of aberrant GnRH neuron function in an animal model of epilepsy. Recordings of GnRH neuron firing and excitability were made in acute mouse brain slices prepared two months after intrahippocampal injection of kainate (KA) or control saline, a well-established TLE model in which most females develop comorbid estrous cycle disruption. GnRH neurons from control females showed elevated firing and excitability on estrus compared with diestrus. By contrast, cells from KA-injected females that developed prolonged, disrupted estrous cycles (KA-long) showed the reverse pattern. Firing rates of cells from KA-injected females that maintained regular cycles (KA-regular) were not different from controls on diestrus, but were reduced on estrus. In KA-injected males, only GnRH neurons in the medial septum displayed elevated firing. In contrast to the diestrus versus estrus and sex-specific changes in firing, GnRH neuron intrinsic excitability was elevated in all KA-injected groups, indicating a role for afferent synaptic and neuromodulatory inputs in shaping overall changes in firing activity. Furthermore, KA-injected females showed cycle-stage-specific changes in circulating sex steroids on diestrus and estrus that also differed between KA-long and KA-regular groups. Together, these findings reveal that the effects of epilepsy on the neural control of reproduction are dynamic across the estrous cycle, distinct in association with comorbid estrous cycle disruption severity, and sex-specific.

## Significance Statement

People with epilepsy are at higher risk of reproductive endocrine disorders compared with the general population, but the neural mechanisms linking epilepsy and these comorbidities are unknown. Here, we report changes in the function of gonadotropin-releasing hormone (GnRH) neurons, which control fertility, in a mouse model of temporal lobe epilepsy (TLE). GnRH neurons from epileptic female mice showed changes in activity dependent on estrous cycle stage and associated with severity of cycle disruption. The impacts of epilepsy on GnRH neurons in males were less severe. These findings provide novel evidence for impacts of epilepsy on GnRH neuron function, and will thus be of clinical relevance in developing new strategies to ameliorate reproductive comorbidities and to treat the underlying seizures and epilepsy.

## Introduction

Reproductive endocrine disorders are prominent comorbidities of epilepsy (Herzog et al., 1986a,b; Bilo et al., 2001; Klein et al., 2001; Löfgren et al., 2007; Bauer and Cooper‐Mahkorn, 2008). For example, 10–20% of women with epilepsy develop polycystic ovary syndrome, in comparison to 5% of women without epilepsy (Bauer et al., 2000; Herzog, 2008). Disrupted menstrual cycle intervals and amenorrhea are also commonly observed (Herzog et al., 2003; Herzog, 2008; Zhou et al., 2012). Furthermore, semen abnormalities are reported in up to 90% of men with epilepsy (Herzog, 2008), and 10–30% of men with focal epilepsy develop low serum testosterone (T) levels (Talbot et al., 2008). The neural mechanisms linking epilepsy to comorbid reproductive endocrine disorders are unknown.

Temporal lobe epilepsy (TLE) is the most common focal epilepsy in patients of reproductive age (Engel, 2001). Roughly 60% of women with TLE not taking antiepileptic drugs exhibit menstrual disorders, indicating that there is a strong association between TLE seizures and reproductive endocrine dysfunction (Herzog et al., 1986a). Furthermore, seizures are exacerbated at certain phases of the menstrual cycle in ∼40% of women with epilepsy, a pattern termed catamenial epilepsy (Laidlaw, 1956; Herzog et al., 2004). One major type of catamenial epilepsy is characterized by a prolonged period of elevated seizure susceptibility and seizure clustering associated with an inadequate luteal phase within irregular, anovulatory menstrual cycles (Herzog et al., 1997). Therefore, seizure control through the restoration of proper reproductive cyclicity could be a novel therapeutic approach for many women with epilepsy. Understanding the mechanisms underlying epilepsy-induced reproductive endocrine disorders is crucial for the development of new strategies for both reproductive cycle maintenance and seizure management.

The hypothalamic gonadotropin-releasing hormone (GnRH) neurons are the final neural output driving the activity of downstream elements of the hypothalamic-pituitary-gonadal (HPG) axis (Herbison, 2006; Christian, 2017). Because of the difficulty in measuring GnRH directly, luteinizing hormone (LH) is often used as a readout of GnRH secretion; pituitary gonadotrophs secrete bolus pulses of LH in response to pulsatile GnRH stimulation. Altered LH pulse frequency has been reported in both men and women with epilepsy (Herzog et al., 1990; Drislane et al., 1994; Quigg et al., 2002), suggesting epilepsy-induced changes in GnRH release. To date, studies of the impacts of epilepsy on GnRH neurons have been limited to anatomic analysis of GnRH immunoreactivity in animal models, with conflicting results (Amado et al., 1993; Friedman et al., 2002; Fawley et al., 2012). Whether GnRH neuron function is impaired in epilepsy has not been tested directly. Furthermore, it is unknown whether the impacts of epilepsy on GnRH neurons vary with the female reproductive cycle, or are different between males and females.

Multiple rodent models of TLE display disrupted estrous cycles (Amado et al., 1987; Edwards et al., 1999; Scharfman et al., 2008), including the intrahippocampal kainate (KA) mouse model (Li et al., 2017). Here, we studied the impacts of epilepsy on GnRH neuron function in this model of TLE. We assessed the spontaneous firing rate and intrinsic excitability of GnRH neurons in brain slices obtained from females (on diestrus and estrus) and males approximately two months after intrahippocampal injection of KA or control saline. Diestrus and estrus were chosen for examination as these stages are associated with changes in seizure susceptibility in rodents (Finn and Gee, 1994; Maguire et al., 2005) and show the greatest degree of change in this model of TLE (Li et al., 2017). Periodic burst firing may underlie GnRH secretion (Kelly and Wagner, 2002). Therefore, we also analyzed the burst properties of the recorded GnRH neurons. Sex steroids exert potent feedback on GnRH neuron activity (Pielecka et al., 2006; Moenter et al., 2009), and altered serum sex steroid levels were reported in other rodent models of epilepsy (Amado et al., 1987; Amado and Cavalheiro, 1998; Edwards et al., 2000; Scharfman et al., 2008). Therefore, we measured serum progesterone (P_4_) and estradiol (E_2_) in control and KA-injected females, and T in males. Our results indicate that the effects of intrahippocampal KA injection on GnRH neuron activity and excitability are different on diestrus compared with estrus, varied according to the severity of comorbid estrous cycle disruption, and sex-specific.

## Materials and Methods

### Animals

All animal procedures were approved by the Institutional Animal Care and Use Committee of the University of Illinois at Urbana-Champaign. GnRH-tdTomato transgenic mice were bred by crossing GnRH-Cre^+^ females (Yoon et al., 2005; The Jackson Laboratory #021207) and Ai9 males (Madisen et al., 2010; The Jackson Laboratory #007909). Both strains are on the C57BL/6J background. Mice were housed in a standard environment in a 14/10 h light/dark cycle (7:00 P.M. lights off) to promote breeding and estrous cyclicity (Fox et al., 2006), with up to five mice per cage. Genotyping to identify pups expressing the Cre allele was done by PCR of DNA extracted from tail clips collected before postnatal day (P)21 using the following four primer sequences as suggested by The Jackson Laboratory: (1) transgene reverse CGG ACA GAA GCA TTT TCC AG; (2) transgene forward ACA GGT GTC TGT CCC ATG TCT; (3) internal positive control forward CAA ATG TTG CTT GTC TGG TG; (4) internal positive control reverse GTC AGT CGA GTG CAC AGT TT.

### Estrous cycle monitoring

A regular mouse estrous cycle is typically 4–5 d long (Byers et al., 2012). Because mouse estrous cycles can be disrupted easily by environmental or other stressors, we categorized cycles up to 6 d in length as “regular” to account for minor temporal disruption and to minimize false positives of estrous cycle disruption. To confirm that all female mice had regular estrous cycles, daily vaginal smears were performed between 10:00 A.M. and 12:00 P.M. starting on or after P42. A total of 20 μl of sterile PBS was gently inserted into the vaginal cavity using a 100- to 200-μl sterile pipette tip, quickly withdrawn, and examined on a microscope slide by brightfield microscopy. Regular estrous cycles were defined as at least two cycles 4–6 d in length with proestrus, estrus, metestrus, and diestrus I/II stages occurring in chronological order. Smears were classified into each stage based on the following criteria: (1) proestrus: dominated by nucleated epithelial cells; (2) estrus: dominated by cornified epithelial cells; (3) metestrus: both cornified epithelial cells and leukocytes; (4) diestrus I: dominated by leukocytes; (5) diestrus II: few or no cells present. Mice that did not display regular estrous cycles within three weeks of monitoring were excluded from further study.

After intrahippocampal injection of either saline or KA, mice were allowed to rest undisturbed for one month to minimize stress and avoid disruption of epileptogenesis. Daily estrous cycle monitoring was then performed until the time of brain slice preparation. The vaginal smears from KA-injected females did not show major changes in smear cytology characteristics. Therefore, the same criteria were used to classify estrous cycle stages for all mice. To promote cyclicity both before and after saline/KA injection, soiled bedding from cages housing male mice was introduced to cages housing females when irregular cyclicity was noted. The average cycle length used to categorize KA-injected mice as “KA-long” (i.e., estrous cycle period ≥7 d) or “KA-regular” was calculated from the daily monitoring for the time from 42 d after injection to the day of brain slice preparation. Cycle period was chosen as the primary parameter for characterization because elongation of the estrous cycle is a prominent feature of cycle disruption in this model of TLE (Li et al., 2017).

### Intrahippocampal injections

Stereotaxic injections in mice eight weeks of age and older were performed under 2–3% oxygen-vaporized isoflurane anesthesia (Clipper Distributing Company). KA (Tocris Bioscience; 50 nl of 20 mM prepared in 0.9% sterile saline) was injected into the right dorsal hippocampal CA1 region (coordinates: 1.8 mm posterior and 1.5 mm lateral to bregma; 1.5 mm ventral to the cortical surface). Control mice were injected with an equivalent volume of sterile saline. Carprofen (5 mg/kg, Zoetis) was administered subcutaneously at the beginning of surgery for analgesia. After closing the scalp incision with sutures, anesthetic 2.5% lidocaine + 2.5% prilocaine cream (Hi-Tech Pharmacal) and Neosporin antibiotic gel (Johnson and Johnson) were applied to the wound.

### Video monitoring of acute seizures

After intrahippocampal injection surgery was completed, mice were placed in a transparent and warmed recovery chamber. All KA-injected mice were video monitored to screen for the development of acute seizures within 5 h after unilateral KA injection. Behavioral seizures of Racine stage 3 (forelimb clonus) and higher (rearing and falling) could be distinguished through the video, whereas behavioral seizures below stage 3 (slight head nodding and facial muscle contraction) could not. Freezing or continuous back-circling behaviors indicating nonconvulsive status epilepticus, as previously reported in this model (Bouilleret et al., 1999; Riban et al., 2002), were also noted.

### Brain slice preparation

Acute brain slices were prepared approximately two months after saline/KA injection. All mice were euthanized by decapitation between 10:00 and 11:00 A.M.; 300-μm coronal brain sections were prepared using a Leica VT1200S (Leica Biosystems) vibrating blade microtome. Brain slices were bathed in oxygenated (95% O_2_, 5% CO_2_) ice-cold sucrose solution (containing 2.5 mM KCl, 1.25 mM NaH_2_PO_4_, 10 mM MgSO_4_, 0.5 mM CaCl_2_, 11 mM glucose, and 234 mM sucrose) during sectioning and then transferred to oxygenated artificial CSF (ACSF) for 30 min at 32°C before being transferred to room temperature for at least 30 min. ACSF contained 2.5 mM KCl, 10 mM glucose, 126 mM NaCl, 1.25 mM NaH_2_PO_4_, 1 mM MgSO_4_, 0.5 mM CaCl_2_, and 26 mM NaHCO_3_; osmolarity ∼298 mOsm. For recording, individual slices were placed in a recording chamber on the stage of an upright BX51WI microscope (Olympus America). Oxygenated bath ACSF was warmed to 30–32°C using an inline heater (Warner Instruments) and pumped through the slice chamber at a flow rate of 2.5 ml/min.

### Targeted extracellular recordings

Targeted extracellular (loose patch) recordings (Nunemaker et al., 2002; Christian et al., 2005) were performed between 11:00 A.M. and 3:00 P.M. for 40–90 min/cell to detect spontaneous firing activity. Thick-walled borosilicate glass recording pipettes (∼2 MΩ tip resistance) were prepared using a P-1000 electrode puller (Sutter Instruments) and filled with filtered ACSF solution with 10 mM HEPES buffer added. GnRH neurons expressing tdTomato red fluorescence were identified by brief illumination at 593 nm and targeted for recording under differential infrared contrast optics using an sCMOS camera (Orca-Flash 4.0LT, Hamamatsu Photonics). Seal resistance was measured at least every 30 min. The initial seal resistances ranged from 3.2 to 12 MΩ and the maximum seal resistance was 45 MΩ. Recordings were performed in voltage-clamp mode with the holding potential at 0 mV and Bessel-filtered at 12 kHz. No more than three cells were recorded per animal. If a cell displayed no action currents within 1 h of recording, 15 mM KCl was bath-applied to induce firing and confirm successful recording. A picture of the pipette tip position was captured using HCImage software (Hamamatsu) after every recording for neuron location analysis. Classification of soma position in the medial septum (MS), preoptic area (POA), or anterior hypothalamic area (AHA) was based on a mouse brain atlas (Paxinos and Franklin, 2012; corresponding plates: MS = 23-25, POA = 25-28, AHA = 29). Data acquisition was performed with a MultiClamp 700B amplifier, Digidata 1550 digitizer, and Clampex 10 software (Molecular Devices). Action current detection was performed using Clampfit 10.6.

### Current-clamp recordings

Recordings were performed in the presence of ionotropic GABA and glutamate receptor blockers added to the bath solution (5 μM APV + 20 μM DNQX + 100 μM picrotoxin, Abcam). Recording pipettes (3–5 MΩ) were filled with a pipette internal solution containing 125 mM K-gluconate, 20 mM KCl, 10 mM HEPES, 4 mM EGTA, 4 mM Mg-ATP, 0.4 mM Na-GTP, and 0.1 mM CaCl_2_; pH 7.2, osmolarity 290 mOsm. After achieving the whole-cell configuration using conventional procedures, a 5-mV depolarizing step from -70 mV holding potential was delivered in voltage-clamp mode to measure series and input resistances. Only recordings with series resistance <20 MΩ and input resistance >500 MΩ were included in analyses. Resting membrane potential was maintained near -73 mV (calculated after correction for a 13-mV liquid junction potential) by applying injected current as needed. All recordings were made in bridge-balanced mode. Data acquisition was performed as for targeted extracellular recordings. Data analysis was performed in Clampfit 10.6 for all parameters except action potential (AP) threshold, which was determined in MATLAB (MathWorks) from the value on the phase plot where the dV/dt was ≥5 V/s. The area under the frequency-current (F-I) curve for each neuron was calculated by a trapezoidal method.

### Firing pattern categorization and burst analysis

Spike train and burst detection analyses were performed in MATLAB. Firing patterns from targeted extracellular recordings were analyzed by constructing an interspike interval (ISI) joint scatter plot, which used the log ISI before (*x*-axis, logISIn) and after (*y*-axis, logISIn + 1) a spike to reveal the temporal relationship of neural spikes (Ramcharan et al., 2000; Dodla and Wilson, 2010). For group comparisons of burst properties, 100 bursts were randomly selected from each neuron to construct cumulative probability distributions. For recordings with fewer than 100 bursts, all detected bursts were used.

### Code accessibility

The code used for GnRH neuron firing pattern recognition, burst detection, burst properties analysis, and statistical comparisons of burst activities is available on Github (https://github.com/ChristianLabUIUC/BurstAnalysis). All code is also available as Extended Data Figure 1.

Extended Data Figure 1. GnRH neuron firing pattern and burst properties analyzer. This code was created in MATLAB R2015b running in a Windows 10 operating system. Run PlotBursts.m in MATLAB to generate the ISI scatter plot, find the optimal burst ISI threshold, view the ratio of data points between quadrants in the scatter plot, generate cumulative probability plots of the examined burst properties, and execute Kolmogorov–Smirnov comparisons of the probability plots. Example data from two GnRH neurons (saline-injected females, diestrus, and estrus) are provided for demonstration purposes. The code is written to analyze raw data containing neuron spike times in the .mat format. More details are available in the README file and documentation within the script. Download Extended Data F, ZIP file.

### Cresyl violet and glial fibrillary acidic protein (GFAP) staining of hippocampus

At the conclusion of brain slice preparation, the remaining portion of cerebrum containing the hippocampus was collected, fixed in 4% PFA for 24 h at 4°C, and preserved in 30% sucrose solution with 0.5% sodium azide at 4°C until sectioning; 50-μm coronal hippocampal sections were prepared using a freezing microtome (SM 2010R, Leica Biosystems). Four to eight sections per mouse from the dorsal hippocampal region were used for verification of hippocampal sclerosis by cresyl violet staining and gliosis by GFAP staining. For cresyl violet staining, sections were mounted on charged glass slides, stained with cresyl violet (Sigma C5042) for 12 min at room temperature (∼22°C), dehydrated with graded ethanol solutions (70–100%), and cleaned in xylene. For GFAP immunostaining, floating sections were incubated in an anti-GFAP mouse monoclonal antibody (1:1000, Sigma G3893) for 48 h at 4°C on a shaker, followed by incubation in Fluorescein horse anti-mouse secondary antibody (1:1000, Vector Laboratories FI-2000) for 2 h at room temperature on a shaker. Sections were then mounted on charged glass slides and coverslipped using Vectashield Hardset Antifade Mounting Medium with DAPI (Vector Laboratories H-1500). Image acquisition was performed using a BX43 light and fluorescence microscope (Olympus) equipped with a Q-Color 3 camera and QCapture 6 software (QImaging).

### Hormone assays

Trunk blood samples from mice used for *in vitro* recordings (*n* = 85 mice) were collected at the time of brain slice preparation. Samples from mice not used for recordings (*n* = 12 mice) were collected after decapitation to replace samples from recorded mice that were contaminated or otherwise unsuitable for analysis. The blood samples were kept at room temperature (∼22°C) for 20 min and then on ice for 20 min, followed by centrifugation at room temperature for 15 min. Serum was withdrawn after centrifugation and stored at -20°C until use. ELISAs (P_4_: DRG Diagnostics; E_2_: Calbiotech; T: IBL America) were performed according to the manufacturers’ instructions. Some samples were diluted to match the volume required for the testing. Samples were run in duplicate and the average of the duplicate was used as the final hormone concentration value for each mouse.

### Experimental design and statistics

**Figure 1.**
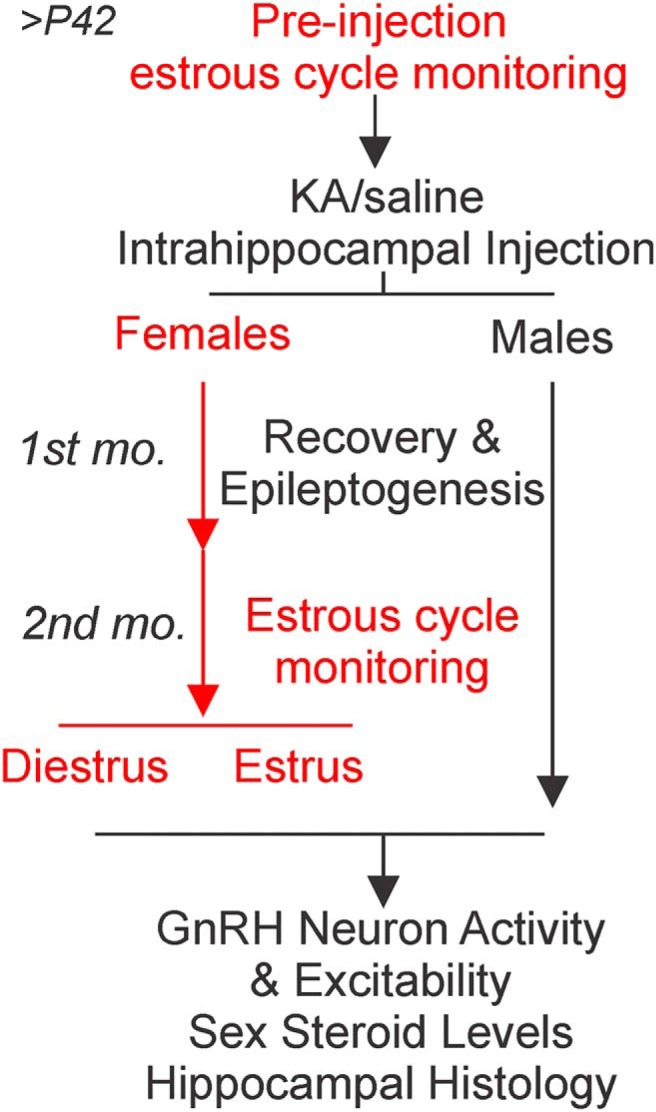
Experimental design and timeline illustrating paradigm of test groups, procedures, and experimental time points. Procedures exclusive to experiments in females are marked in red.

The experimental design is outlined in Figure 1. Pre-injection estrous cycle monitoring for adult female mice started on or after P42. Age-matched female and male mice were stereotaxically injected with saline or KA in the dorsal hippocampus. Two months after injection, acute brain slices were prepared and GnRH neuron firing rate and excitability were measured via single-cell electrophysiological recordings. For female mice, recordings were performed on the days of diestrus or estrus. At the time of brain slice preparation, trunk blood serum and hippocampal tissue were collected for hormone analysis and histology, respectively.

Statistical comparisons were made using OriginPro (OriginLab), SPSS (IBM), or R software. Comparisons between two groups (e.g., diestrus vs estrus within each treatment group, and saline vs KA for males) were made using *t* tests or Mann–Whitney tests depending on the normality of the data, which was determined using Shapiro–Wilk tests. Comparisons between saline, KA-long, and KA-regular females were made separately for diestrus and estrus using Kruskal–Wallis and Dunn’s *post hoc* tests or one-way ANOVA and Bonferroni *post hoc* tests based on the normality and the homogeneity of variance within each group as assessed by Shapiro–Wilk tests and Levene’s tests, respectively. Data for evoked firing rate and excitability parameters were Box-Cox transformed to achieve normal distributions, and analyzed using three-way ANOVA and Fisher’s LSD *post hoc* tests. Comparisons of P_4_ and E_2_ levels between saline, KA-long, and KA-regular groups at each cycle stage were made using one-way ANOVA and Fisher’s *post hoc* tests; non-normally distributed data were normalized by log-transformation before analysis. Results in the above tests are reported as means ± SEM. Firing patterns of GnRH neurons from female mice were assessed using a logistic regression with treatment group (saline, KA-long, and KA-regular) and estrous cycle stage (diestrus and estrus) as factors. The probabilities of neurons displaying bursting, irregular, tonic, or quiet firing patterns were analyzed separately using Fisher’s exact tests. Comparisons were made between treatment groups within diestrus or estrus, or within treatment groups between diestrus and estrus. χ^2^ tests were used to compare the proportions of GnRH neurons from control and KA-injected male mice that showed bursting and irregular firing patterns, and to compare the proportions of KA-long and KA-regular mice showing hippocampal sclerosis. Correlation analysis was performed using Spearman’s rank-order tests. Statistical significance in the above statistical tests was set at *p* < 0.05. Cumulative probability distributions were compared using Kolmogorov–Smirnov goodness-of-fit tests, and the criterion for statistical significance in these tests was *p* < 0.001.

## Results

### Confirmation of hippocampal KA injection targeting

Mice treated with intrahippocampal KA exhibit acute non-convulsive or mild clonic status epilepticus followed (within two weeks to two months) by spontaneous focal seizures that rarely generalize to tonic-clonic seizures, along with histopathological features including hippocampal sclerosis and gliosis, recapitulating cardinal hallmarks of human TLE (Bouilleret et al., 1999; Riban et al., 2002; Blümcke et al., 2013). In these studies, we applied three steps of verification to check the accuracy of intrahippocampal injections: (1) video screening of seizures immediately after KA injection; (2) histopathological assessment of hippocampal sclerosis by cresyl violet (Nissl) staining; and (3) immunofluorescent GFAP staining for gliosis. The small subset of mice that were used for trunk blood collection only, without *in vitro* recordings, all displayed acute seizures following KA injection. Therefore, hippocampal tissue used for histology was collected on the day of brain slice preparation for *in vitro* recordings (approximately two months after injection).

**Figure 2.**
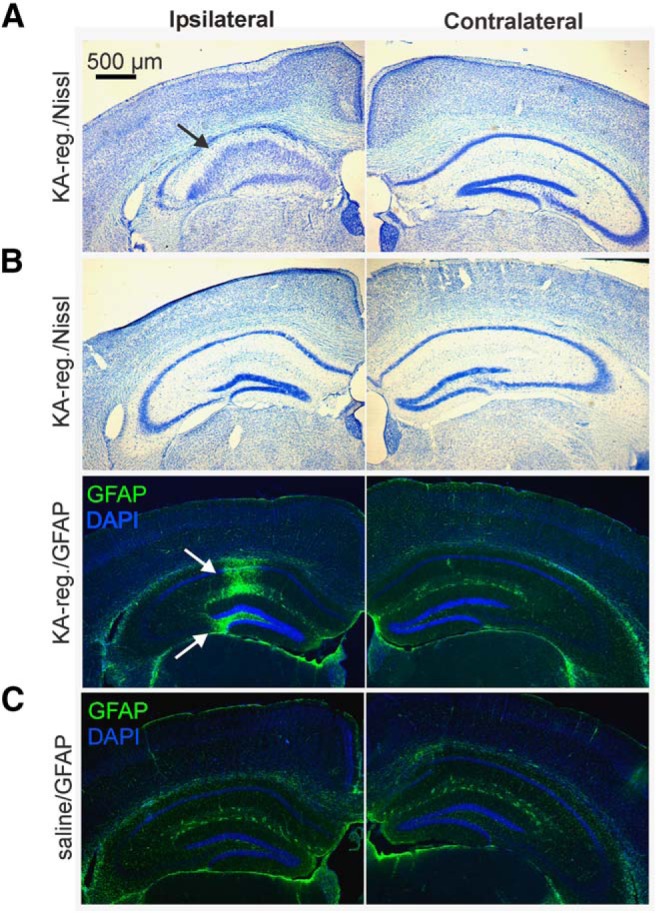
Verification of KA injection targeting. ***A***, Example cresyl violet staining from a KA-regular female with marked granule cell dispersion ipsilateral to the injection, and intact hippocampus contralateral to the injection. ***B***, Cresyl violet (top) and GFAP/DAPI staining (bottom) from a KA-regular female. Note the strong GFAP immunoreactivity in the injected hippocampus, despite absence of major pathology observed in cresyl violet staining of adjacent sections. GFAP, green; DAPI, blue. Left, Ipsilateral to the injection. ***C***, Example GFAP staining in tissue from a saline-injected mouse. Scale bar: 500 μm. Black arrow, hippocampal sclerosis detected by cresyl violet stain; white arrows, gliosis in CA and dentate gyrus detected by GFAP staining.

The large majority of KA-injected female mice (57 of 69, 83%) showed at least two seizures within 5 h after KA injection, and five mice showed one seizure. Of the remaining seven mice, three stayed frozen or exhibited backwards circling over the entire recording, one mouse did not show seizures or frozen/backwards circling behavior after KA injection, and three were not successfully video recorded (Table 1). Four of these seven mice displayed prominent granule cell dispersion and other signs of sclerosis in cresyl violet staining, as shown in the example in Figure 2*A*. Hippocampi that showed no obvious signs of sclerosis were subsequently evaluated for GFAP staining. In two of the remaining three mice examined, gliosis was observed in the dentate gyrus and/or CA regions in the injected hippocampus, as shown in the example in Figure 2*B*. The remaining KA-injected female, which did not show acute seizures, hippocampal sclerosis, nor gliosis, was excluded from the final dataset. No signs of sclerosis or gliosis were observed in the dentate gyrus or CA regions collected from randomly selected saline-injected mice (Fig. 2*C*). Neither gliosis nor hippocampal sclerosis were observed in the contralateral hippocampus.

**Table 1.** Outcomes of video screening of acute seizures and hippocampal histology to verify KA injection targeting

Video screening outcome (acute seizures)				
	>2 seizures	1 seizure	No seizures or video	Total
Females	57	5	7	69
Males	10	0	5	15
Histology for mice with no seizures or video (2 months post-KA injection)				
	Sclerosis	Gliosis	No sclerosis or gliosis	Total
Females	4	2	1*	7
Males	5	0	0	5

Number of seizures refers to behavioral seizures (Racine stage 3 or higher) observed within 5 h of KA injection. Hippocampal tissue from mice that either did not show acute seizures or for whom videos were not available was collected approximately two months after KA injection. Sclerosis was assessed via cresyl violet (Nissl) staining. Sections of hippocampi that did not display signs of sclerosis were further assessed for gliosis via GFAP staining; *, mouse removed from dataset in absence of either video confirmation of acute seizure induction or later development of hippocampal sclerosis/gliosis.

Video screening of 13 KA-injected males confirmed that 10 (77%) showed at least two seizures (Table 1). The remaining three mice did not show any behavioral seizures but were confirmed to have developed hippocampal sclerosis by two months after injection. Two other male mice were not successfully video recorded, but were confirmed by cresyl violet staining to have developed hippocampal sclerosis.

### Rate of development of hippocampal sclerosis does not correlate with severity of comorbid estrous cycle disruption

A subset of KA-injected females can maintain regular estrous cyclicity through two months after injection (Li et al., 2017). This resilience may reflect inaccuracy of the initial injection or reduced induction of hippocampal damage. Therefore, the hippocampi of all KA-injected females that maintained regular 4- to 6-d estrous cycles (KA-regular) were examined by histology, even if acute seizures were detected in the videos. Hippocampi of 12 out of the 20 mice (60%) in this group showed granule cell dispersion in cresyl violet staining (Fig. 2*A*). Seven of the remaining eight mice showed gliosis with GFAP staining (Fig. 2*B*). The remaining mouse was not confirmed by histology due to problems with tissue sectioning, but remained in the final dataset as it was confirmed to have shown acute seizures.

To determine whether the presentation of sclerosis was higher in mice that developed prolonged estrous cycle lengths (≥7 d period, KA-long), we evaluated the hippocampi of 25 randomly selected KA-long mice. Nineteen of these mice (76%) showed sclerosis in cresyl violet staining; this proportion was not different from that of the KA-regular group (*p* > 0.6, χ^2^ test). These results indicate that the severity of comorbid estrous cycle disruption following KA injection is not directly correlated with the rate of induction of hippocampal sclerosis, and that downstream changes in the HPG axis likely play a significant role in driving the comorbidity.

### GnRH neurons from KA-injected female mice show altered firing rates on diestrus and estrus

**Figure 3.**
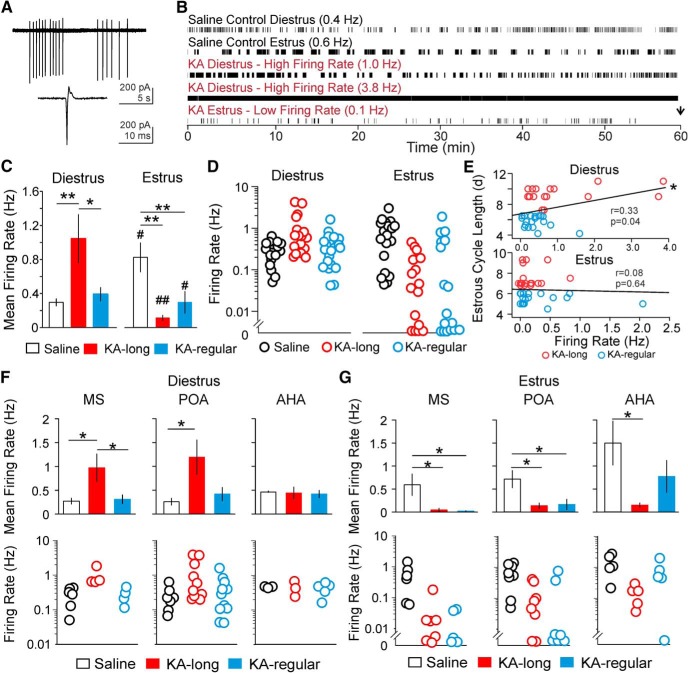
Diestrus versus estrus shifts in GnRH neuron mean firing rate are compromised in the intrahippocampal KA mouse model of TLE. ***A***, Example raw traces of bursts (top) and individual (bottom) action currents detected in loose patch recordings. ***B***, Representative raster plots of activity in GnRH neurons from control and KA-injected females. The black arrow marks the end of recording. The mean firing rate of each cell is given in parentheses. ***C***, Mean ± SEM for GnRH neuron firing rate in control (open bars), KA-long (red bars), and KA-regular (blue bars) groups. KA-injected females are divided into KA-long and KA-regular groups based on their estrous cycle length (KA-long ≥ 7 d, KA-regular 4–6 d). Cells were recorded on diestrus (left) or estrus (right). ***D***, Firing rates in individual cells, plotted on a logarithmic scale to show the full range. ***E***, Correlation analyses between GnRH neuron firing rate and estrous cycle length in KA-injected females performed with data combined from KA-long (red circles) and KA-regular (blue circles) groups. Black line, line of best fit for all points. ***F***, ***G***, Comparison of GnRH neuron firing rate between controls, KA-long, and KA-regular groups based on anatomic location of somata for cells recorded on diestrus (***F***) or estrus (***G***). Data are shown as group mean firing rates (top, mean ± SEM) and individual neuron firing rates (bottom); *p < 0.05, **p < 0.01 for comparisons between saline, KA-long, and KA-regular females by Kruskal–Wallis with Dunn’s *post hoc* tests; #*p* < 0.05, ##*p* < 0.01 for comparisons between diestrus and estrus within groups by *t* tests or Mann–Whitney tests. In scatter plots of individual neuron firing rate, neurons plotted below y = 0.01 showed firing rates ≥0 Hz and below 0.01 Hz.

For studies of female mice, acute coronal brain slices were prepared on either diestrus or estrus approximately two months after intrahippocampal saline/KA injection. Targeted extracellular loose patch recordings were performed to detect spontaneous action currents, the fast currents underlying APs (Fig. 3*A*), in GnRH neurons. All GnRH neurons recorded were ipsilateral to the injected hippocampus. tdTomato^+^ somata in the MS, POA, and AHA displaying the bipolar morphology typical of GnRH neurons were targeted for recording. GnRH neurons from control mice typically showed firing activity with periodic quiescence between groups (bursts) of action currents (Fig. 3*B*). Cells recorded on estrus had higher mean firing rates than cells recorded on diestrus (diestrus *n* = 16 cells/8 mice, estrus *n* = 20 cells/9 mice; *p* = 0.03; Mann–Whitney test; Fig. 3*C*,*D*), providing novel evidence for an endogenous diestrus-to-estrus shift in mean firing rate in control conditions.

By contrast, GnRH neurons from KA-injected females showed abnormal firing patterns (Fig. 3*B*). On diestrus, GnRH neurons from KA-long mice (*n* = 17 cells/9 mice) showed an increase in mean firing rate compared with both control (*n* = 16 cells/8 mice, *p* = 0.01) and KA-regular mice (*n* = 20 cells/10 mice, *p* = 0.016; Kruskal–Wallis test/Dunn’s). The mean firing rate of GnRH neurons from KA-regular females was not different from controls (Fig. 3*C*). GnRH neurons from KA-long females showed high firing rates ≤3.8 Hz, although firing rates for some cells fell within the normal range (Fig. 3*D*). When the data from KA-long and KA-regular females were combined, there was a positive linear correlation between firing rate and cycle length on diestrus (*r* = 0.33, *p* = 0.043, Spearman’s rank-order test; Fig. 3*E*). These results suggest that the impacts of KA injection on GnRH neuron firing activity on diestrus are correlated with the severity of comorbid estrous cycle disruption.

On estrus, GnRH neurons from both KA-long (*n* = 18 cells/8 mice, *p* = 0.01) and KA-regular (*n* = 17 cells/7 mice, *p* = 0.01) females showed significantly lower firing rates compared with controls (*n* = 20 cells/9 mice; Fig. 3*C*, Kruskal–Wallis test/Dunn’s). Some cells from KA-long and KA-regular females showed only a few or no APs (Fig. 3*D*). GnRH neuron firing rate and estrous cycle length were not correlated on estrus (*r* = 0.082, *p* = 0.64, Spearman’s test; Fig. 3*E*). Therefore, in contrast to the results obtained on diestrus, the impacts of KA injection on the firing activity of GnRH neurons on estrus are similar in mice with and without comorbid estrous cycle disruption. Moreover, the KA-induced impact on activity of GnRH neurons from KA-long mice is opposite that observed on diestrus.

Notably, in contrast to the typical elevation in firing on estrus compared with diestrus in control mice, the diestrus-to-estrus shift in firing was reversed in KA-long mice, with decreased firing on estrus compared with diestrus (*p* < 0.0001; Mann–Whitney test). Although mean firing rates of cells from KA-regular mice were similar between diestrus (0.39 ± 0.08 Hz) and estrus (0.29 ± 0.13 Hz), a nonparametric Mann–Whitney test revealed a significant difference (*p* = 0.03), which appeared to be driven mainly by a subset of neurons with firing rates close to zero on estrus (Fig. 3*D*). These results suggest that KA injection disrupts the normal patterns of diestrus versus estrus changes in GnRH neuron firing activity.

### GnRH neuron location influences firing rate response to KA injection

To determine whether the firing rate phenotype of each cell was influenced by the soma location, the recorded GnRH neurons were classified based on the location of the recording pipette tip in the MS, POA, or AHA. Although this analysis necessitated parsing the overall data into more groups, some of which only had a few cells, distinct patterns emerged based on the anatomic classification.

On diestrus, GnRH neurons in the MS from KA-long females (*n* = 4 cells/4 mice) showed higher firing rates than cells from controls (*n* = 6 cells/3 mice, *p* = 0.034) and KA-regular mice (*n* = 4 cells/4 mice, *p* = 0.043, Kruskal–Wallis test/Dunn’s). GnRH neurons in the POA from KA-long females (*n* = 10 cells/7 mice) had higher firing rates than neurons from controls (*n* = 7 cells/6 mice, *p* = 0.025), and a borderline level of significance in comparison to neurons from KA-regular mice (*n* = 11 cells/9 mice, *p* = 0.053, Kruskal–Wallis test/Dunn’s). Firing rates of cells in the AHA, however, were not different between the three groups (saline *n* = 3 cells/3 mice, KA-long *n* = 3 cells/3 mice, KA-regular *n* = 5 cells/5 mice, *p* > 0.8; Fig. 3*F*).

On estrus, GnRH neurons in the MS and POA from both KA-long (*n* = 5 cells/4 mice MS, *n* = 8 cells/7 mice POA) and KA-regular (*n* = 5 cells/5 mice MS, *n* = 7 cells/6 mice POA) females displayed decreased firing rates compared to controls (*n* = 6 cells/5 mice MS, *n* = 8 cells/6 mice POA; MS: KA-long vs saline *p* = 0.019, KA-regular vs saline *p* = 0.042; POA: KA-long vs saline *p* = 0.037, KA-regular vs saline *p* = 0.045, Kruskal–Wallis/Dunn’s). Cells in the AHA from KA-long females recorded on estrus (*n* = 5 cells/5 mice) showed decreased firing compared with controls (*n* = 6 cells/5 mice; KA-long vs saline *p* = 0.027; Kruskal–Wallis test/Dunn’s), but cells from KA-regular mice (*n* = 5 cells/4 mice) were not different from controls (Fig. 3*G*). Together, these results indicate that, on both diestrus and estrus, firing rates of GnRH neurons in the MS and POA are most strongly affected following KA injection. On estrus, however, AHA cells also appear to be affected in mice with the most severe comorbid estrous cycle disruption.

### GnRH neuron firing patterns are altered following KA injection in female mice

**Figure 4.**
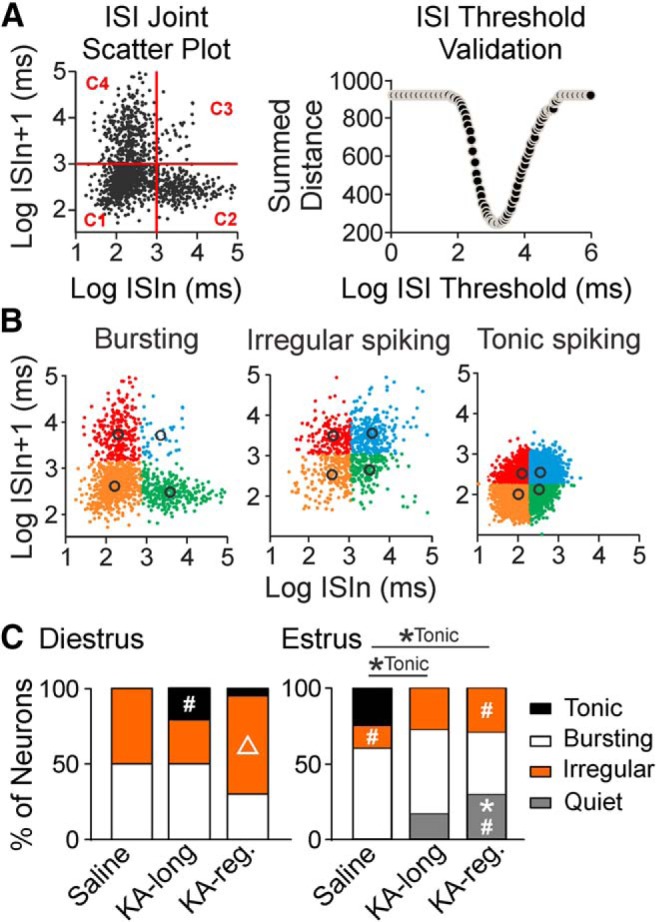
GnRH neuron firing patterns are altered in KA-injected female mice on both diestrus and estrus. ***A***, ***B***, Examples of burst detection and firing pattern categorization. ***A***, left, Example ISI joint scatter plot with a randomly selected candidate burst ISI threshold value (red line). The four quadrants divide all data into four clusters: C1, C2, C3, and C4. Right, Example ISI threshold validation shows the summed distance for each candidate burst ISI threshold value. The summed distance is calculated by the summation of squared distance between every point and its corresponding cluster centroid. The candidate value with the smallest summed distance is chosen as the optimal burst ISI threshold. ***B***, Examples of scatter plots for GnRH neuron bursting (left), irregular spiking (middle), and tonic spiking (right) patterns. The different colors represent the final C1 to C4 distribution with the optimal burst ISI threshold for each cell. Black circles, individual centroids of clusters C1–C4. ***C***, Proportion of GnRH neurons from female mice categorized into each pattern on diestrus (left) and estrus (right); *p < 0.05 for pair-wise Fisher’s exact test comparisons for indicated firing pattern between control and KA-injected groups; #*p* < 0.05 for comparisons for indicated firing pattern between diestrus and estrus within control and KA-injected groups. Δ, *p* < 0.05 for comparisons for indicated firing pattern between KA-long and KA-regular groups within the same estrous cycle stage.

The full spike train of each recorded GnRH neuron was used to categorize firing patterns by constructing ISI joint scatter plots. The scatter plots were divided into four quadrants (clusters) by a series of candidate burst ISI threshold values. The optimal value among the candidate burst ISI thresholds was determined as the value at which the degree of proximity was highest for all four clusters, quantified as the intersection point of the cluster limit lines producing the lowest squared summed distance between all points within a cluster and its respective centroid (center of the cluster; Fig. 4*A*).

In accordance with other studies of GnRH neuron firing pattern *in vitro* and *in vivo* (Moenter et al., 2003; Constantin et al., 2013), the neurons recorded in this dataset showed bursting, irregular spiking, or tonic spiking patterns (Fig. 4*B*). When a neuron shows a bursting pattern, each quadrant (C1–C4) on the ISI scatter plot contains a cluster that encompasses the spikes within bursts, at the beginning of each burst, at the end of each burst, and outside the burst, respectively. In bursting neurons, the spikes in cluster C1 (lower left quadrant) outnumber other clusters, producing a skewed distribution of ISI values across clusters. The number of spikes to define a burst was set more than or equal to four. Therefore, for a cell to be identified as a bursting neuron, the C1 cluster needed to contain at least two times as many points as C2 (lower right) and C4 (upper left), and at least five times as many points as C3 (upper right) in the ISI joint scatter plot. When a neuron showed an irregular or tonic spiking pattern, the ISI values were divided almost equally across the four quadrants. Neurons were classified as tonic spiking when the centroids for all clusters were located at ISI values <1 s.

The results of logistic regression analysis for each firing pattern examining effects of KA injection, cycle stage, and interaction between KA injection and cycle stage are summarized in Table 2. For burst firing, there were no effects of KA injection or cycle stage, and no interaction. For irregular spiking, there was an effect of cycle stage, but no effect of KA injection and no interaction. For tonic spiking, there were no effects of cycle stage or KA injection separately, but there was a significant interaction. For quiet cells, there were effects of both KA injection and cycle stage, but no interaction.

**Table 2.** Effects of KA injection, estrous cycle stage, or an interaction between KA injection and cycle stage on probability of occurrence of each firing pattern in logistic regression analysis

Firing pattern	KA injection	Cycle stage	Interaction of KA injection and cycle stage
Tonic	0.17	0.84	0.0009***
Bursting	0.18	0.30	0.99
Irregular	0.15	0.0072**	0.25
Quiet	0.027*	0.0003***	1.00

*p* values from logistic regressions performed for each firing pattern; *p < 0.05, **p < 0.01, ***p < 0.001.

Group-specific differences for irregular, tonic, and quiet activity were further analyzed *post hoc* using pair-wise Fisher’s exact tests (Fig. 4*C*). Cells from saline-treated controls showed a greater proportion of cells showing irregular spiking on diestrus compared with estrus (*p* = 0.034). Analysis of tonic spiking showed a borderline significant difference between diestrus and estrus (*p* = 0.053). Cells from KA-long mice showed more tonic firing on diestrus than on estrus (*p* = 0.045), and cells from KA-regular mice showed more irregular spiking on diestrus than on estrus (*p* = 0.049) and more quiescence on estrus than on diestrus (*p* = 0.014).

On diestrus, no differences were detected between KA-injected groups and controls for any firing pattern, but comparisons between the KA-long and KA-regular groups found that cells from KA-regular mice showed increased irregular spiking (*p* = 0.049), with no differences detected for tonic firing or quiescence. On estrus, cells from control mice showed more tonic firing in comparison to both KA-long (*p* = 0.048) and KA-regular (*p* = 0.049) groups. Cells from KA-regular mice also showed increased quiescence compared with controls (*p* = 0.014). No differences were detected between KA-long and KA-regular groups on estrus. On diestrus, the tonic-spiking neurons from KA-long females were in the POA (three neurons) and MS (one neuron), and the one tonic-spiking neuron from a KA-regular female was in the POA. On estrus, the tonic-spiking neurons from controls were in the AHA (three neurons), POA (one neuron), and MS (one neuron), and quiet cells from both KA-long and KA-regular females were in the POA and MS (KA-long: two POA, one MS; KA-regular three POA, two MS). Bursting and irregular-spiking cells in all groups were evenly distributed across the MS, POA, and AHA. These results indicate that following KA injection, a subset of GnRH neurons in the MS and POA displays a continuous tonic firing activity pattern on diestrus that is not observed in controls. Furthermore, in stark contrast to the firing patterns observed on diestrus, subsets of GnRH neurons in the MS and POA show aberrant quiescence on estrus after KA injection.

### GnRH neuron burst properties are altered following KA injection in female mice

To assess whether GnRH neuron burst firing (which may be linked to hormone release) is altered after KA injection, the cells that were categorized as “bursting” were further examined for detailed analysis of burst properties. Note that because bursting cells were distributed across all three anatomic areas examined, location categories were collapsed for this analysis.

**Figure 5.**
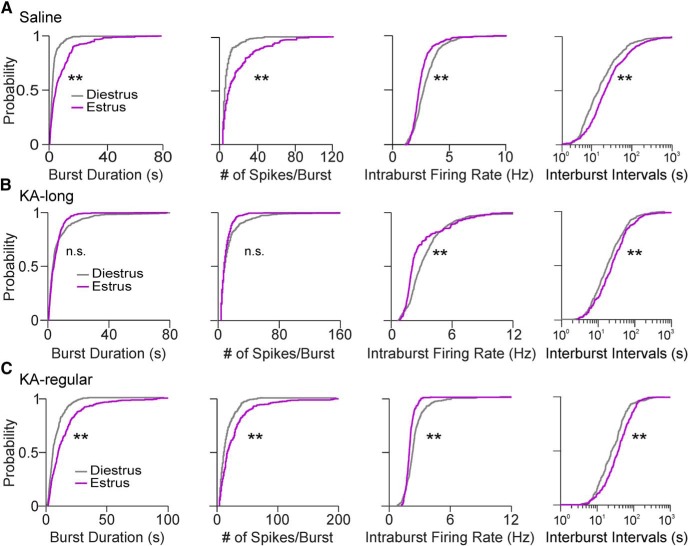
GnRH neuron burst properties on diestrus and estrus; only neurons displaying burst spiking patterns were used for comparisons. ***A***, Cumulative probability distributions for burst properties of GnRH neurons from control female mice on diestrus (gray traces) and estrus (purple traces). Cumulative distributions were constructed using 100 randomly selected bursts per cell. ***B***, Burst properties from KA-long female mice. ***C***, Burst properties from KA-regular female mice; **p < 0.0001 for comparisons by Kolmogorov–Smirnov tests. n.s., not significant. The interburst intervals are presented on logarithmic scales for better visualization of the major portion (1–99%) of the distributions.

GnRH neurons from control mice showed distinct burst properties between diestrus and estrus. Bursting cells showed longer burst duration, more spikes per burst, and slower intraburst firing rate on estrus compared with diestrus (diestrus *n* = 8 cells, estrus *n* = 12 cells; all *p* < 0.0001, pairwise Kolmogorov–Smirnov tests). The intervals between bursts were also longer on estrus than on diestrus (*p* < 0.0001; Fig. 5*A*). GnRH neurons from KA-long females did not display the difference in burst duration and number of spikes per burst between cycle stages, but intraburst firing rate was decreased and interburst interval was longer on estrus than on diestrus (diestrus *n* = 10 cells, estrus *n* = 10 cells; both *p* < 0.0001, pairwise Kolmogorov–Smirnov tests; Fig. 5*B*). GnRH neurons from KA-regular females showed the same directions of change as controls (diestrus *n* = 6 cells, estrus *n* = 5 cells), with increased burst duration, number of spikes per burst, and interburst interval, as well as decreased intraburst firing rate, on estrus compared with diestrus (all *p* < 0.0001, pairwise Kolmogorov–Smirnov tests; Fig. 5*C*). These results demonstrate that GnRH neuron burst properties fluctuate with the estrous cycle, showing changes indicative of increased bursting (and potentially increased hormone release) on estrus compared with diestrus.

**Figure 6.**
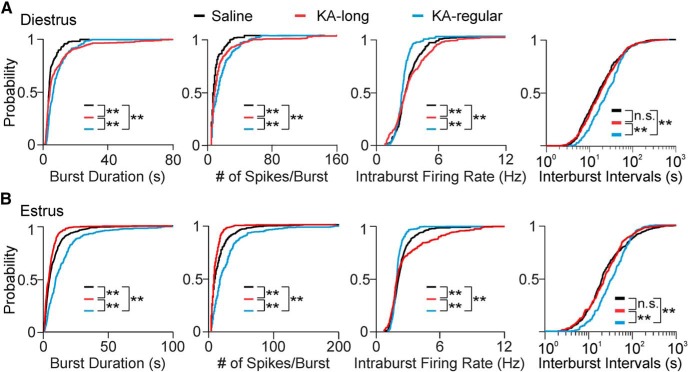
Bursting GnRH neurons from KA-injected female mice show changed burst properties. ***A***, Cumulative probability distributions for burst properties in cells displaying bursting patterns from control (black traces), KA-long (red traces), and KA-regular (blue traces) mice recorded on diestrus. Cumulative distributions were constructed using 100 randomly selected bursts per cell. ***B***, Cumulative probability distributions for burst properties recorded on estrus; **p < 0.01 for comparisons between saline, KA-long, or KA-regular groups by pairwise Kolmogorov–Smirnov tests. n.s., not significant. The interburst intervals are presented on logarithmic scales for better visualization of the major portion (1–99%) of the distributions.

On diestrus, cells from KA-injected mice showed increased burst duration and number of spikes per burst compared with controls (saline *n* = 8 cells; KA-long *n* = 8 cells; KA-regular *n* = 6 cells; pairwise Kolmogorov–Smirnov tests: KA-long vs saline: *p* < 0.0001, KA-regular vs saline: *p* < 0.0001; Fig. 6*A*). The distributions of values for KA-long and KA-regular groups, however, were also distinct from each other, with the KA-long group showing increased probability of the longest burst durations and highest numbers of spikes per burst (*p* < 0.0001). In comparison to controls, cells from KA-long females showed higher intraburst firing rates (*p* < 0.0001), but conversely, cells from KA-regular females showed decreased intraburst firing rates (*p* < 0.0001). In addition, the intervals between bursts were prolonged in GnRH neurons from KA-regular females compared with controls (*p* = 0.0006; Fig. 6*A*), but cells from KA-long females did not show this difference. These latter findings of decreased intraburst firing rate and increased interburst interval in cells from KA-regular mice may represent compensatory mechanisms engaged to decrease burst-driven GnRH release in this group.

On estrus, GnRH neurons from KA-long females displayed shorter bursts, fewer spikes per burst, and higher intraburst firing rates than controls (saline *n* = 12 cells; KA-long *n* = 10 cells; all *p* < 0.0001, pairwise Kolmogorov–Smirnov tests). GnRH neurons from KA-regular females (*n* = 5 cells), however, showed changes in the opposite direction, with longer burst duration, more spikes per burst, and slower intraburst firing rate than controls (all *p* < 0.0001). GnRH neurons from KA-regular females also showed an altered distribution of interburst interval values compared to those from KA-long females and controls (*p* < 0.0001; Fig. 6*B*). These findings indicate that, although overall firing rates between cells from KA-long and KA-regular females are similar on estrus, the burst properties are quite different. Moreover, the burst properties of cells from KA-regular mice on estrus show the same pattern of differences compared with controls as observed on diestrus. For cells from KA-long mice, however, the effects on burst duration and number of spikes per burst on estrus in comparison to controls are opposite those observed on diestrus.

### GnRH neuron intrinsic excitability changes from diestrus to estrus and is persistently increased in KA-injected female mice

**Figure 7.**
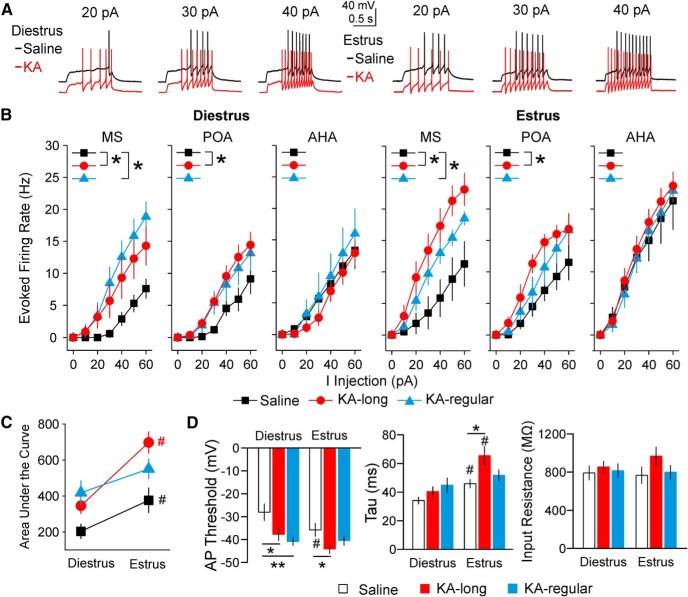
GnRH neuron intrinsic excitability is elevated on both diestrus and estrus in the intrahippocampal KA mouse model of TLE. ***A***, Representative examples of evoked firing in response to depolarizing current steps in cells recorded on diestrus (left) and estrus (right). The KA traces are offset to highlight differences in spiking. All traces started from a membrane potential of approximately -73 mV, corrected for the liquid junction potential. ***B***, Frequency-current (F-I) curves for GnRH neurons recorded on diestrus (left) or estrus (right), classified by the location of the somata of recorded neurons. Depolarizing current steps were applied in increments of 10 pA; *p < 0.05 for comparisons of area under the curve by three-way ANOVA with Fisher’s LSD. ***C***, Mean ± SEM for area under the curve of evoked firing rate plots on diestrus and estrus in cells from control (black symbols and line), KA-long (red symbols and line), and KA-regular (blue symbols and line) mice. ***D***, Mean ± SEM for AP threshold, membrane time constant (τ), and input resistance; *p < 0.05, **p < 0.01 by two-way ANOVA with Fisher’s LSD; #*p* < 0.05 for comparisons between diestrus and estrus within groups by three-way ANOVA with Fisher’s LSD.

We used whole-cell current-clamp recordings to determine whether the observed changes in firing activity are associated with changes in intrinsic excitability. Three-way ANOVA showed treatment group, estrous cycle stage, and soma location all had effects on the GnRH neuron evoked firing rate (treatment group and estrous cycle: *p* < 0.001, soma location *p* = 0.019). Neurons in the control group had higher evoked firing rates on estrus than on diestrus (diestrus = 18 cells/8 mice, estrus = 20 cells/5 mice, *p* = 0.005, three-way ANOVA/Fisher’s LSD; Fig. 7*A–C*), indicating an endogenous increase in excitability on estrus compared with diestrus in control conditions.

Because there was no interaction between treatment group and estrous cycle stage (*p* = 0.13, overall three-way ANOVA), the data from diestrus and estrus were initially combined to examine the overall effects of KA injection on GnRH neuron excitability. In this analysis, GnRH neurons from both KA-long and KA-regular females showed increased excitability compared with controls (saline *n* = 38 cells/13 mice, KA-long *n* = 41 cells/13 mice, KA-regular *n* = 30 cells/11 mice; saline vs KA-long, *p* < 0.001, saline vs KA-regular, *p* = 0.001; Fig. 7*B*,*C*). Furthermore, comparisons made examining each cycle stage separately identified elevated excitability in both KA-injected groups compared with controls on both diestrus and estrus (diestrus: saline *n* = 18 cells/8 mice, KA-long *n* = 26 cells/8 mice, KA-regular *n* = 15 cells/7 mice; saline vs KA-long *p* = 0.047, saline vs KA-regular *p* = 0.008, Fisher’s LSD; estrus: saline *n* = 20 cells/5 mice, KA-long *n* = 15 cells/5 mice, KA-regular *n* = 15 cells/4 mice; saline vs KA-long *p* < 0.001, saline vs KA-regular *p* = 0.045, Fisher’s LSD). These results suggest that GnRH neuron intrinsic excitability is elevated in mice both with and without comorbid estrous cycle disruption after KA injection, and that this higher excitability persists across both diestrus and estrus.

Fisher’s LSD *post hoc* tests were used to examine the differences between control and KA-injected groups with respect to soma location. Specifically, GnRH neurons from KA-long females in MS and POA, but not in AHA, showed increased excitability compared with controls at both cycle stages (MS *p* = 0.048 diestrus, *p* < 0.001 estrus; POA *p* = 0.021 diestrus, *p* = 0.027 estrus; *n* = 5–13 cells for each group and location). Only GnRH neurons in MS from KA-regular females showed increased excitability compared with controls at both cycle stages (diestrus *p* = 0.003, estrus *p* = 0.027; *n* = 5–9 cells for each location). No difference was observed between KA-long and KA-regular groups in either MS or POA, and no effect of KA injection was observed in the AHA region (Fig. 7*B*). These results suggest that, as with the effects on overall firing rate, GnRH neurons in the MS and POA show the greatest change in excitability after KA injection.

To investigate whether KA-injected females also showed cycle-stage-dependent fluctuations in GnRH neuron excitability as observed in the control mice, evoked firing rates from KA-injected mice were compared between diestrus and estrus. Cells from KA-long females showed higher excitability on estrus compared with diestrus (*p* < 0.001). Cells from KA-regular females showed a similar trend but not to the level of significance (*p* = 0.067; Fig. 7*C*). Together with the changes in burst firing specific to the KA-regular group described above, this trend may also represent a compensatory mechanism to bring intrinsic excitability values closer to the control range, particularly on estrus.

Additional excitability parameters were analyzed to evaluate potential mechanisms of increased GnRH neuron evoked firing rate observed in cells from KA-injected female mice. These parameters included AP threshold, input resistance, capacitance, membrane time constant (τ), latency to firing, ISI, and instantaneous frequency. Because cells in the MS and POA, but not AHA, showed changes in evoked firing rate, these excitability parameters were analyzed for MS and POA cells only. Three-way ANOVA showed that the soma location did not affect any of the tested parameters. Therefore, the data from both MS and POA were grouped together for further analysis. On diestrus, neurons from KA-long and KA-regular females showed hyperpolarized AP threshold compared with controls (*p* = 0.021 KA-long vs saline, *p* < 0.001 KA-regular vs saline, two-way ANOVA/Fisher’s LSD). On estrus, neurons from KA-long, but not KA-regular, females showed hyperpolarized AP threshold (*p* = 0.038, two-way ANOVA/Fisher’s LSD) and increased τ (*p* = 0.006, two-way ANOVA/Fisher’s LSD) compared with controls (Fig. 7*D*). No differences were seen in input resistance or the other parameters (Fig. 7*D*; Table 3). None of the AP kinetics parameters examined (full-width at half-maximum, afterhyperpolarization amplitude, time to afterhyperpolarization, rise slope and decay slope) were different between control and KA-injected groups (Table 3). Together, these results indicate that the observed changes in overall intrinsic excitability are manifest most notably in hyperpolarized AP threshold on both diestrus and estrus, and increased membrane time constant on estrus. Furthermore, these effects are most prominent in GnRH neurons from KA-injected mice with more severe estrous cycle disruption.

**Table 3.** GnRH neuron excitability parameters and AP kinetics for each treatment and cycle stage in females

Parameters	Diestrus			Estrus			Overall ANOVA (F value)	
	Saline	KA-long	KA-reg.	Saline	KA-long	KA-reg.	Treatment	Cycle Stage
AP threshold (mV)	-28 + 3.6	-39.1 + 2.5*	-40.8 + 1.7**	-35.8 + 2.8^#^	-44.0 + 2.0*	-40.4 + 2.1	6.57*	4.54*
Input resistance (MΩ)	791.5 + 73.0	852.3 + 62.3	812.8 + 75.3	766.8 + 85.9	965.0 + 97.1	796.0 + 73.1	1.37	0.43
Capacitance (pF)	18.5 + 2.7	14.8 + 0.7	16.5 + 1.6	17.9 + 2.0	16.1 + 0.8	18.5 + 1.1	0.63	1.21
τ (ms)	34.1 + 2.4	40.4 + 3.4	44.7 + 5.2	45.9 + 2.9^#^	65.3 + 6.6*^#^	51.7 + 3.9	4.45*	17.02**
Latency to firing (ms)	0.57 + 0.07	0.47 + 0.05	0.48 + 0.08	0.50 + 0.07	0.36 + 0.08*	0.43 + 0.06	1.66	2.40
ISI first 10 spikes (ms)	10.0 + 0.3	9.1 + 0.2	9.6 + 0.4	9.1 + 0.4	9.8 + 0.5	9.2 + 0.3	2.71	0.50
Ins. freq. first 10 spikes (Hz)	112.9 + 6.1	122.3 + 4.1	117.9 + 6.0	130.3 + 7.2	113.8 + 5.7	119.8 + 5.1	0.10	0.50
FWHM (ms)	2.1 + 0.1	2.2 + 0.1	2.3 + 0.2	2.4 + 0.1	2.5 + 0.2	2.4 + 0.1	0.99	0.43
AHP (pA)	35.0 + 3.2	29.7 + 2.0	27.9 + 1.8	30.2 + 1.4	28.7 + 2.0	27.9 + 2.0	2.52	0.33
Time to AHP (ms)	3.8 + 0.1	3.8 + 0.1	3.7 + 0.2	4.1 + 0.2	3.9 + 0.3	3.8 + 0.3	0.19	0.76
Max rise slope	171.3 + 11.7	182.7 + 12.6	155.8 + 14.4	155.5 + 12.2	157.1 + 13.0	163.1 + 15.4	0.58	1.66
Max decay slope	-80.9 + 3.7	-78.8 + 4.2	-67.4 + 6.5	-74.8 + 3.8	-71.9 + 3.4	-73.2 + 3.8	1.94	0.88

ISI first 10 spikes, average ISI of the first 10 evoked spikes; Ins. freq. first 10 spikes, average instantaneous frequency of the first 10 evoked spikes; FWHM, full-width at half-maximum; AHP, afterhyperpolarization; time to AHP, time between the AP initiation and the peak of AHP; *p < 0.05, **p < 0.01 two-way ANOVA with Fisher’s LSD *post hoc* tests.

Comparisons of GnRH neuron excitability parameters within groups between diestrus and estrus also indicated differences. AP threshold was hyperpolarized on estrus compared with diestrus in control mice (*p* = 0.022), but not in KA-long or KA-regular mice (KA-long *p* = 0.063, KA-regular *p* = 0.9); τ was increased on estrus compared with diestrus in control and KA-long mice (saline *p* = 0.013, KA-long *p* < 0.001), but not in KA-regular mice (*p* = 0.12; Fig. 7*D*). These results indicate that KA-injected groups do not display the typical diestrus-to-estrus difference in AP threshold observed in controls. In addition, a lack of diestrus versus estrus difference in membrane time constant may represent another compensatory mechanism specific to the KA-regular group.

### Changes in circulating P_4_ and E_2_ levels two months after KA injection in females

**Figure 8.**
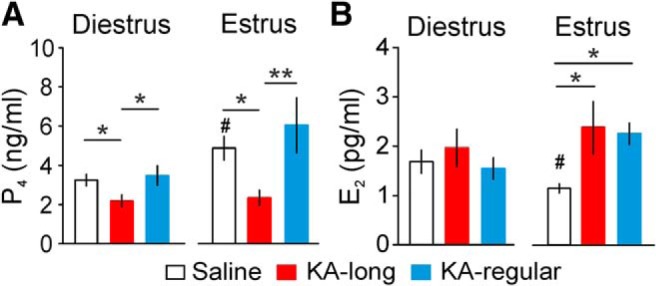
Changes in circulating P_4_ and E_2_ levels on diestrus and estrus as measured two months after KA injection. ***A***, Mean ± SEM for P_4_ levels on diestrus (left) and estrus (right) in control (open bars), KA-long (red bars), and KA-regular (blue bars) mice. ***B***, Mean ± SEM for E_2_ levels on diestrus (left) and estrus (right); *p < 0.05 for comparisons between saline, KA-long, and KA-regular groups by one-way ANOVA and Fisher’s *post hoc* tests; #*p* < 0.05 for comparisons between estrus and diestrus within groups by *t* tests.

Changes in GnRH neuron activity could impact downstream gonadal function, including production and secretion of sex steroids. Reciprocally, sex steroid feedback can act at the hypothalamic level to affect GnRH neuron activity, and at the hippocampal level to modulate seizure susceptibility. Therefore, to determine whether circulating levels of the female sex steroid hormones P_4_ and E_2_ are altered in the intrahippocampal KA mouse model of TLE, we assayed trunk blood serum by ELISA. In controls, P_4_ levels were higher on estrus than on diestrus (diestrus = 23 mice, estrus = 18 mice, *p* = 0.01, two-sample *t* test). In KA-long and KA-regular mice, P_4_ levels were not significantly different between diestrus and estrus (KA-long diestrus *n* = 19 mice, estrus *n* = 10 mice, *p* > 0.6; KA-regular diestrus *n* = 20 mice, estrus *n* = 7 mice, *p* = 0.14). Serum P_4_ levels were reduced in KA-long females compared with both controls and KA-regular females on both diestrus (KA-long vs saline *p* = 0.042, KA-long vs KA-regular *p* = 0.02; one-way ANOVA/Fisher’s LSD) and estrus (KA-long vs saline *p* = 0.036, KA-long vs KA-regular *p* = 0.007; one-way ANOVA/Fisher’s LSD). P_4_ levels in KA-regular females were not different from controls at either cycle stage (*p* > 0.3; Fig. 8*A*). These results indicate that suppression of P_4_ levels on both diestrus and estrus is associated with increased severity of comorbid estrous cycle disruption after KA injection.

In control mice, serum E_2_ levels were lower on estrus than diestrus (diestrus = 6 mice, estrus = 6 mice, *p* = 0.035, two-sample *t* test). In KA-long and KA-regular mice, the E_2_ levels were not significantly different between diestrus and estrus (KA-long diestrus *n* = 8 mice, estrus *n* = 10 mice *p* = 0.59; KA-regular diestrus *n* = 6 mice, estrus *n* = 5 mice *p* = 0.07). On diestrus, E_2_ levels were not different between the three groups, but on estrus E_2_ levels were significantly higher in KA-injected females compared with controls (KA-long vs saline *p* = 0.036, KA-regular vs saline *p* = 0.016; one-way ANOVA/Fisher’s LSD), with no difference observed between KA-long and KA-regular females (Fig. 8*B*). These results indicate that KA-injected mice both with and without comorbid estrous cycle disruption lack the typical decrease in E_2_ levels on estrus compared with diestrus.

### Male mice show modest disruption of GnRH neuron activity after KA treatment without changes in T levels

**Figure 9.**
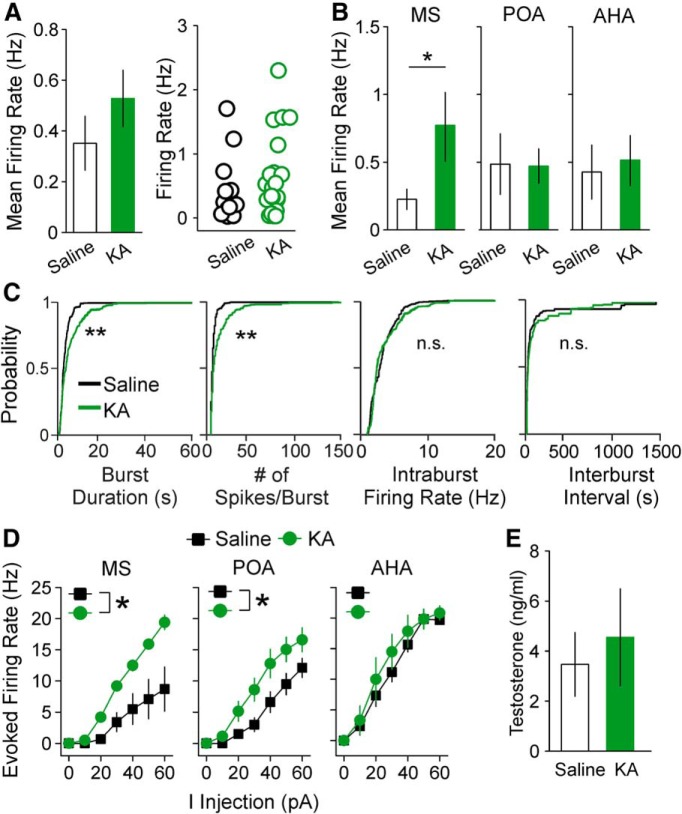
Impacts of KA injection on GnRH neuron mean firing rate and excitability in male mice depend on soma location. ***A***, Mean ± SEM for mean firing rate (left) and firing rates for individual GnRH neurons (right) from males treated with saline (open bars and circles) or KA (green bars and circles). ***B***, Mean ± SEM for mean firing rate of GnRH neurons from control and KA-injected males classified by soma location; *p < 0.05, two-sample *t* test. ***C***, Cumulative probability distributions for burst duration, number of spikes per burst, intraburst firing rate, and interburst intervals in cells from control and KA-injected males; **p < 0.0001 by Kolmogorov–Smirnov tests. ***D***, F-I curves for GnRH neurons from control and KA-injected males; *p < 0.05 for comparison of area under the curve by two-way ANOVA with Fisher’s LSD *post hoc* tests. ***E***, Mean ± SEM for serum T in control and KA-injected male mice.

An advantage of integrating mouse models of TLE with GnRH-tdTomato mice is that we can also assess impacts of epilepsy on GnRH neurons from male mice, which lack a parameter akin to the estrous cycle that can be used as a high-throughput assay of reproductive endocrine comorbidities. To determine whether the effects of KA treatment on GnRH neurons are sex-specific, we measured firing rate, burst properties, and intrinsic excitability of GnRH neurons from control and KA-injected male mice at two months after surgery. There was not a change in overall mean firing rate between KA-injected and control groups (saline *n* = 18 cells/10 mice, KA *n* = 25 cells/13 mice, *p* = 0.14; Fig. 9*A*), but comparison of mean firing rates based on soma location revealed that MS GnRH neurons from KA-injected males displayed increased firing compared with controls (saline *n* = 5 cells, KA *n* = 7 cells, *p* = 0.034, Mann–Whitney test; Fig. 9*B*). Burst properties were altered, with longer burst duration and increased number of spikes per burst observed in the KA-injected group (saline *n* = 5 cells, KA *n* = 6 cells, both *p* < 0.0001, pairwise Kolmogorov–Smirnov tests; Fig. 9*C*). The saline-injected mice had bursting GnRH neurons in the POA (four neurons) and AHA (one neuron), whereas the KA-injected mice had bursting GnRH neurons in the POA (five neurons) and MS (one neuron). The intraburst firing rate and interburst interval were not different between control and KA-injected groups (Fig. 9*C*), and the proportion of GnRH neurons from male mice showing irregular spiking or bursting was not affected by KA treatment (control: 61% irregular and 39% bursting; KA-injected: 72% irregular and 28% bursting; *p* > 0.5, χ^2^ test). No cells showed tonic spiking or quiet patterns. These results suggest that the impacts of KA injection on GnRH neuron firing are different in males compared with females.

In whole-cell current-clamp recordings, GnRH neurons in the MS and POA from KA-injected males showed higher evoked firing rates compared with controls (MS *p* = 0.028, POA *p* = 0.044, two-way ANOVA/Fisher’s LSD; *n* = 5–10 cells for each group and location; Fig. 9*D*). Cells in the AHA did not show a KA-induced difference in excitability. No other parameters of excitability and AP kinetics were different between control and KA-injected males (Table 4). These results indicate that, as observed in females, excitability of MS and POA GnRH neurons is elevated in KA-injected males, although the change in excitability is limited to evoked firing rate. In addition, serum T levels in KA-injected males were not different from controls (Fig. 9*E*).

**Table 4.** GnRH neuron excitability parameters and AP kinetics for saline and KA-injected males

Parameters	Saline	KA
AP threshold (mV)	-40.6 + 2.9	-42.3 + 2.3
Input resistance (MΩ)	745.6 + 36.9	1892.2 + 984.2
Capacitance (pF)	16.7 + 1.0	15.7 + 1.0
τ (ms)	51.0 + 3.7	61.7 + 6.5
Latency to firing (ms)	628.1 + 67.2	493.5 + 68.6
ISI first 10 spikes (ms)	123.4 + 5.4	121.4 + 3.7
Ins. freq. first 10 spikes (Hz)	9.9 + 1.2	9.9 + 0.5
FWHM (ms)	2.3 + 0.12	2.4 + 0.3
AHP	31.6 + 2.6	32.8 + 1.9
Time to AHP	3.6 + 0.1	3.5 + 0.2
Max rise slope	187.3 + 11.7	158.9 + 3.2
Max decay slope	-77.5 + 5.1	-75.3 + 4.2

ISI first 10 spikes: average ISI of the first 10 evoked spikes; Ins. freq. first 10 spikes, average instantaneous frequency of the first 10 evoked spikes; FWHM, full-width at half-maximum; AHP, afterhyperpolarization; time to AHP, time between the AP initiation and the peak of AHP. Two-sample *t* tests for each parameter did not identify any differences between controls and KA-injected groups.

## Discussion

Pathologies in the neural control of reproduction likely link epilepsy and comorbid reproductive endocrine disorders, but specific functional changes in key neuronal populations regulating reproduction and fertility, including GnRH neurons, have not been described. The present studies provide direct evidence for changes in GnRH neuron function with epilepsy, and indicate that impacts on GnRH neuron function are associated with severity of comorbid estrous cycle disruption, different on diestrus compared with estrus, and sex-specific. Overall, GnRH neurons from KA-injected diestrous females, estrous females, and males shared some commonalities as well as some group-specific changes. GnRH neurons in the MS and POA showed increased evoked firing rates across all groups, but only females showed significantly hyperpolarized AP threshold. In addition, these results are the first to show that unilateral KA injection alters circulating sex hormone levels in female mice. Due to the complex feedback loops involved, it remains difficult at present to distinguish changes that are direct consequences of epileptiform activity in the hippocampus from those that are secondary alterations that develop as a feedback response to comorbid estrous cycle disruption. The changes common to all groups, however, likely reflect the direct consequences of KA-induced epilepsy.

### Linking hippocampal seizure activity to changes in hypothalamic GnRH neuron function

Seizures in TLE originate primarily in the hippocampus and rarely become generalized (Engel, 1996). The intrahippocampal KA mouse model of TLE similarly shows recurrent spontaneous paroxysmal discharges mainly restricted to the vicinity of the injected hippocampal area (Riban et al., 2002), although recent work using cortical surface recordings in the same model detected generalized spikes propagating across frontal cortices as well (Sheybani et al., 2018). Whether there is a correlation between hippocampal seizure burden and temporal proximity of recent seizure activity to GnRH neuron functional abnormalities and estrous cycle disruption will be investigated by incorporating electroencephalogram recordings in future studies. In this regard, spontaneous seizures in the intrahippocampal KA mouse model are present by approximately two weeks after KA injection (Riban et al., 2002; Heinrich et al., 2011), whereas the estrous cycle disruption in this model does not emerge until approximately six weeks after KA injection (Li et al., 2017). This time course indicates that the robust pattern of disrupted estrous cyclicity develops gradually after epilepsy is fully established, perhaps reflecting a cumulative effect of seizure burden over time (Raedt et al., 2009). This temporal sequence should provide important insights in future studies about the causal relationships between epileptogenesis, seizure severity, GnRH neuron pathology, and estrous cycle disruption.

Focal hippocampal seizures could potentially affect GnRH neurons through direct and/or indirect projections between the hippocampus and the hypothalamus. Indeed, it has long been known that electrical stimulation in hippocampus can reduce gonadotropin release and prevent ovulation, indicating functional links between the hippocampus and hypothalamic circuits for reproduction (Velasco and Taleisnik, 1969; Gallo et al., 1971). The present data indicate that GnRH neurons with aberrant firing activity in females are primarily located in the MS and POA. Similarly, the MS is the only region that contained GnRH neurons with increased firing rates in male mice, although cells in the POA showed altered bursting properties. There are robust bidirectional projections between the hippocampus and the MS (Gaykema et al., 1991; Mattis et al., 2014). The prominence of KA-induced changes in GnRH neurons located in the MS suggests that these pathways may provide anatomic substrates for seizure activity and/or the secondary consequences of hippocampal epileptiform activity to propagate and impact hypothalamic GnRH neurons. The extent and characteristics of reorganization of hippocampal projections to GnRH neuron circuitry following intrahippocampal KA injection remain unclear. Furthermore, GnRH neurons form a heterogeneous population with variations, for example, in firing properties (Constantin et al., 2013), neuromodulator receptor expression (Jasoni et al., 2005), and participation in the preovulatory GnRH/LH surge (Hoffman et al., 1993; Wintermantel et al., 2006; Christian and Moenter, 2007) associated with differences in soma location. The relationship between the anatomic location of GnRH neuron somata and cellular heterogeneity has not yet been fully characterized, but the present data support a working model in which the location of the GnRH neuron soma helps to shape the functional outcome in the face of epilepsy, in concert with changes in hippocampal-hypothalamic projections differentially targeting the MS, POA, and AHA.

### Pathologic hallmarks of TLE in hippocampus and hypothalamus

Hippocampal sclerosis is a common hallmark of TLE in humans (Margerison and Corsellis, 1966; Cook et al., 1992; Blümcke et al., 2013; Blumcke et al., 2017). The intrahippocampal KA mouse model of TLE reproduces this hippocampal sclerosis in multiple ways, including extensive neuronal loss, gliosis, and hippocampal circuit reorganization. Specifically, the complete degeneration of CA1 and CA3 is often observed in ipsilateral dorsal hippocampus, along with enlargement of the dentate granule cell layer (Bouilleret et al., 1999; Riban et al., 2002; Fig. 2*A*). Although intrahippocampal KA injection induces acute injury and cell loss, it should be noted that estrous cyclicity can persist following complete ablation of the hippocampus in rats (Terasawa and Kawakami, 1973). Furthermore, we observed similar rates of hippocampal sclerosis induction in both KA-long and KA-regular groups. Therefore, the changes observed in GnRH neuron function likely reflect epileptic circuit reorganization and downstream propagating effects of seizure activity, rather than acute effects of the initial precipitating hippocampal injury per se (i.e., effects independent of epileptiform activity). The extent of hypothalamic pathology in the intrahippocampal KA mouse model used here remains unclear. However, neither cell loss nor gliosis were observed in the hypothalamus in a unilateral KA macaque model of epilepsy (Chen et al., 2013), and no GnRH neuron cell loss was observed in a systemic pilocarpine mouse model (Fawley et al., 2012).

### Changes in GnRH neuron firing properties: potential links to downstream HPG axis malfunction

Changes in LH pulse frequency have been reported in women with epilepsy in the absence of antiepileptic drug treatment, even when regular menstrual cyclicity is maintained (Bilo et al., 1991; Meo et al., 1993). Altered GnRH-LH release in patients with epilepsy may thus represent a form of subclinical reproductive dysfunction. Because hormone secretion by neuroendocrine cells, such as vasopressin and oxytocin neurons, has long been linked with burst firing activities (Wakerley and Lincoln, 1973; Dutton and Dyball, 1979), we examined whether KA injection affected GnRH neuron firing patterns and/or burst properties. Although the proportions of cells showing burst firing were not altered following KA injection, there were significant differences in burst properties in cells from both KA-injected male and female mice. Moreover, the changes observed in KA-injected female mice were distinct depending on whether estrous cycles were of regular or long length, indicating that changes in GnRH neuron burst patterning differ with the severity of the comorbidity. High and low GnRH pulse frequency favors the pituitary synthesis and release of LH and follicle-stimulating hormone, respectively (Wildt et al., 1981), but the effect of GnRH pulse duration (perhaps reflected in burst duration) on gonadotropin release is still unknown. In addition, the aberrant presence of tonic spiking neurons on diestrus and quiet neurons on estrus could potentially impair overall GnRH neuron network communication and rhythmicity. In this regard, GnRH neurons express GnRH receptors and the firing activity of GnRH neurons changes in response to application of GnRH (Xu et al., 2004; Todman et al., 2005; Han et al., 2010). At the pituitary level, higher rates of GnRH neuron firing activity may also give rise to sustained elevations in GnRH content in the pituitary vasculature, which could downregulate the pituitary response to GnRH (Belchetz et al., 1978; Smith et al., 1983). Further studies are thus needed to determine whether altered GnRH neuron firing patterns drive dysregulated GnRH and gonadotropin secretion in epilepsy.

On diestrus, there was a moderate but significant correlation between GnRH neuron firing rate and estrous cycle length in KA-injected mice, indicating a relationship between elevated GnRH neuron activity and the severity of the estrous cycle comorbidity. The estrous cycle is regulated by a complex interplay involving hypothalamic GnRH release, pituitary gonadotropin release, and ovarian response. Although it is unknown whether LH or FSH release is altered in this model of epilepsy, the ovaries do not show major histopathological changes at two months after KA injection (Li et al., 2017), indicating that the estrous cycle phenotype is not reflective of gross ovarian damage. Therefore, although it would be premature to directly link the changes in GnRH neuron mean firing rate to the effects on estrous cyclicity, the positive correlation observed indicates that changes in GnRH neuron activity, and potentially downstream pituitary gonadotropin release, are likely to be major players in this comorbidity.

### Relationships of sex steroid feedback and epilepsy-associated changes in GnRH neuron activity

Changes in GnRH neuron firing could be, at least in part, the consequences of altered sex hormone feedback. P_4_ typically exerts strong suppression of GnRH neuron activity (Pielecka et al., 2006; Bashour and Wray, 2012) and GnRH release (Goodman and Karsch, 1980; Leipheimer et al., 1984; He et al., 2017). Our finding of decreased P_4_ levels (and the likely reduction of P_4_ negative feedback) in KA-long females on diestrus is thus consistent with the increased mean firing rate observed in this group. P_4_ levels were also reduced in KA-long females on estrus, but this change is not consistent with the significantly suppressed firing rate of GnRH neurons from this group at this stage, suggesting other mechanisms are likely involved. P_4_ may still play a role in driving suppressed firing in KA-regular females on estrus, however, as this group did not show a reduction in circulating P_4_.

On both diestrus and estrus, E_2_ levels were within the low physiologic range in which E_2_ typically exerts negative (i.e., suppressive) feedback on GnRH release (Sarkar and Fink, 1980; Evans et al., 1994). Control mice had higher E_2_ levels on diestrus compared to estrus. This result is in line with the lower GnRH neuron firing rate on diestrus, although an alternative possibility is that the boost in GnRH neuron firing rate observed on early estrus reflects the tail end of the robust increase in firing activity that drives the preovulatory GnRH surge during late proestrus (Christian and Moenter, 2010). Furthermore, the increased E_2_ levels on estrus in both KA-long and KA-regular females would be predicted to exert increased negative feedback on GnRH neurons, and are thus consistent with the decreased GnRH neuron firing rate observed at this cycle stage in both groups. Serum E_2_ levels on diestrus were similar between KA-injected and control females, suggesting that E_2_ does not contribute to the observed increase in GnRH neuron firing on diestrus in the KA-long group. These differences in circulating P_4_ and E_2_ levels could reflect altered ovarian steroidogenesis and/or compromised metabolism of sex steroids by peripheral cytochrome P450 oxidases (Runtz et al., 2018). Mechanistic investigation of the roles of E_2_ and P_4_ feedback in mediating epilepsy-associated changes in GnRH neuron function will be an important aspect of future study, for example through selective hormone replacement in gonadectomized mice, or incorporation of mouse models in which specific steroid hormone receptors are absent or not functional.

Alterations in circulating steroid levels could also exert impacts at the level of hippocampus to modulate seizure activity. For example, central P_4_ conversion to the neurosteroid allopregnanolone can reduce seizure susceptibility and epileptiform activity through potent positive allosteric modulation of hippocampal GABA_A_ receptors (Maguire et al., 2005; Lawrence et al., 2010; Reddy and Rogawski, 2012). Therefore, reduced P_4_ levels in the KA-long mice may also be indicative of reduced neurosteroid production in the brain, which could exacerbate seizure activity in this group.

In a study examining the systemic pilocarpine model of TLE in female rats, circulating T was elevated concomitant with the presence of cystic ovaries (Scharfman et al., 2008). In the present studies, measurement of T in the females was inconclusive because the serum amounts that remained after completing the assays for P_4_ and E_2_ were too small to be reliably assayed in duplicate. However, it should be noted that many values were below the ELISA detection limit, and those samples that did yield values were not different between control and KA-injected mice (unpublished observations). Together, these results suggest that circulating T was not elevated in the KA-injected female mice. This finding is consistent with previous work in which ovarian cysts were not observed in this mouse model of TLE (Li et al., 2017), and thus elevated T levels would not be expected.

Of note, the pattern of changes in E_2_ and P_4_ levels detected in this mouse model does not directly match changes in sex steroid levels reported in a limited number of human clinical studies (Herzog et al., 2003; Kalinin and Zheleznova, 2007). Human TLE, however, is a more heterogeneous condition than one animal model can reproduce. The location of seizure origin, the effects of different anti-epileptic drugs, and timing of hormone level measurement in relation to menstrual cycle stage could all profoundly influence the phenotype of changes in sex steroid levels induced by epilepsy. Further exploration of neuroendocrine disruption in both the clinic and in preclinical models is needed to elucidate the spectrum of changes that can be induced by epilepsy and seizure activity, and to determine differential mechanisms that may drive these varying outcomes.

### Relative contributions of GnRH neuron intrinsic excitability and synaptic inputs in driving epilepsy-associated changes in GnRH neuron activity

In contrast to the bidirectional changes in GnRH neuron firing rate observed in KA-injected female mice, intrinsic excitability was persistently elevated in KA-injected groups on both diestrus and estrus, and irrespective of estrous cycle period phenotype. Hyperpolarized AP threshold was the most prominent change observed in cells from KA-injected females. Tetrodotoxin-sensitive voltage-gated sodium channels control the AP depolarization in GnRH neurons (Norberg et al., 2013), and therefore may play important roles in determining AP threshold. GnRH neuron AP threshold and latency to firing can also be modulated by E_2_ feedback, for example through A-type potassium currents (DeFazio and Moenter, 2002). Changes in these and other underlying conductances likely shape the overall shifts in excitability produced both across estrous cycle stages and in response to epilepsy. In addition, the persistent increase in excitability of GnRH neurons from KA-injected mice, together with the dynamic changes in mean firing rate, indicate that afferent synaptic and/or neuromodulatory inputs likely play a role in shaping the cycle-stage-dependent shifts in overall firing behavior. In this regard, most E_2_ and P_4_ feedback to GnRH neurons is mediated trans-synaptically (Sullivan and Moenter, 2005; Wintermantel et al., 2006; Christian and Moenter, 2007), and certainly at least one synaptic connection is needed to transmit the effects of hippocampal seizure activity to hypothalamic GnRH neurons. It will be interesting in future studies to determine which steroid hormone-sensitive and hormone-insensitive pathways are involved in transmitting the effects of seizure activity to GnRH neurons, and in driving differential changes dependent on estrous cycle stage.

### Concluding remarks

The present results provide novel direct evidence of aberrant GnRH neuron activity and excitability in an animal model of epilepsy. Most importantly, this study supports a model in which the pattern of changes in GnRH neuron firing activity in epilepsy is not a fixed property, but is profoundly influenced by the overall physiologic state. Furthermore, these studies emphasize the utility of this model of TLE in distinguishing which changes are observed concomitant with estrous cycle disruption, and which changes are observed in resilient females that maintain regular estrous cyclicity. These findings thus have important implications for further studies of the neural mechanisms linking epilepsy to comorbid reproductive endocrine dysfunction, and provide an indication that treatment strategies for both seizures and reproductive comorbidities need to be tailored based on cycle stage, comorbidity severity, and sex.

## Synthesis

Reviewing Editor: Quentin Pittman, University of Calgary Decisions are customarily a result of the Reviewing Editor and the peer reviewers coming together and discussing their recommendations until a consensus is reached. When revisions are invited, a fact-based synthesis statement explaining their decision and outlining what is needed to prepare a revision will be listed below. The following reviewer(s) agreed to reveal their identity: Aristea Galanopoulou, Jamie Maguire.

The manuscript entitled “Dynamic and Sex-Specific Changes in Gonadotropin-Releasing Hormone Neuron Activity and Excitability in a Mouse Model of Temporal Lobe Epilepsy” is an original research manuscript addressing a novel and largely unexplored topic with translational relevance. This topic will be of interest to the field, but may be limited in regards to broader readership interest. This manuscript extensively studies changes in GnRH neuronal activity in a mouse model of temporal lobe epilepsy with or without estrous cycle disruption. The authors employ an efficient experimental plan to examine neuropathological features of epileptogenesis, estrous cycle disruption, GnRH activity, and circulating hormone levels. This manuscript is largely descriptive, characterizing a model of estrous cycle disruption in a mouse model of temporal lobe epilepsy, but provides limited mechanistic information regarding estrous cycle disruption in epilepsy. This is the first preclinical study to demonstrate direct evidence for changes in GnRH function associated with epilepsy, which is a significant contribution to the field. The study is well done, despite the unavoidable limitations, most of which are well discussed and comments are to further clarify some aspects of the study:

1.One limitation of this study is that it is unclear whether cycle disruption is related to seizure frequency/severity or temporal proximity to a previous seizure event or ictal activity. As it appears that EEG (seizure activity) was not monitored prior to slice collection this should be raised as a possibility; if seizure activity has been monitored this should be reported.

2. The authors should discuss the relevance of the effect of differences in GnRH activity relative to location (MS and POA vs. AHA).

3. The authors should also discuss what is known regarding the firing patterns required for GnRH function. What is the physiological relevance of these changes in firing patterns? The hormonal measurements are interesting, especially the lower E2 levels is estrus than diestrus, which might be related to the timing of sampling. A discussion on how these hormonal levels compare with other studies in mice would be useful. For the particular study however, it would be worth looking whether there is any correlation between the GnRH firing properties and patterns and the hormones (P4, E2 and/or ratio) for each mouse, regardless of the estrous cycle stage.

4. The authors should mention the criteria they use for staging. This is because there is some variance in the way progesterone and estradiol levels in diestrus and estrus are reported across estrous cycle stages in the literature and here. For example, in some studies estradiol is reported higher in estrus than in diestrus and the reverse for progesterone (see Wood et al 2007 in Reproduction), while others agree more with the authors. So it would be appropriate to clarify the criteria and design and perhaps comment on whether such criteria allowed clear differentiation of stages, particularly when cycle was disturbed and where hormonal levels were measured.

5. The authors should briefly discuss possible mechanisms underlying the changes in intrinsic excitability and firing patterns of GnRH neurons in KA-treated mice. What underlies the change in AP threshold?

6. The author suggest that the changes in GnRH function may result from the observed changes in estradiol and progesterone levels. The authors should consider experimentally testing this hypothesis, which could contribute more mechanistic information regarding changes in GnRH excitability and cycle disruption in epilepsy.

7. The authors should consider discussing how the observed hormone changes relate to the human condition of neuroendocrine disruption in epilepsy patients.

8. The authors could discuss the implications regarding the evidence of epileptogenesis occurring by 2 weeks whereas the cycle disruption is not evident until 6 weeks.

9.The model was reproduced as right hippocampal KA injection. Why was the right side chosen? Was there any pathology on the left hippocampus in any of the mice with estrous cycle abnormalities? In humans it has been proposed that the left TLE is more often associated with hypothalamic / gonadal dysfunction. So that would be an interesting point to discuss for the pathology of the current study and consider for future studies

10. There is no word on hypothalamic pathology and how this correlates with the current results. It would be important for the authors to comment since they must have such data.

Minor:

1.Please included time of day when sampling was done.

2. Significance statement: “PWE are higher risk of reproductive endocrine abnormalities”. Compared to whom? The authors should clarify,

3. Why a 14:10 light/dark cycle was chosen?

4. The current terms are ‘focal epilepsies’ not partial. Please correct.

5. Page 14: “rarely generate to tonic clonic seizures”: perhaps change to “rarely generalize to tonic-clonic seizures”

6.The authors should attempt to make the manuscript more concise. The manuscript is quite long and may be a bit much to tackle for those outside this very specialized field.

7.The discussion is largely a summary of the results. The discussion could be reworked to discuss the translational relevance of the findings, mechanistic underpinnings, and broader implications.

6.The authors state that the results “indicate that afferent synaptic and/or neuromodulatory inputs likely play a large role in shaping the cycle-stage-dependent shifts in overall firing behavior. While this is true, the intrinsic excitability changes are also interesting and deserve further attention.

## Author Response

We thank the reviewers and the editor for their helpful comments on our manuscript. In response, we have made several edits, particularly to the Discussion, and added information to the Methods and Results sections as requested.

Critique:

The manuscript entitled “Dynamic and Sex-Specific Changes in Gonadotropin-Releasing Hormone Neuron Activity and Excitability in a Mouse Model of Temporal Lobe Epilepsy” is an original research manuscript addressing a novel and largely unexplored topic with translational relevance. This topic will be of interest to the field, but may be limited in regards to broader readership interest. This manuscript extensively studies changes in GnRH neuronal activity in a mouse model of temporal lobe epilepsy with or without estrous cycle disruption. The authors employ an efficient experimental plan to examine neuropathological features of epileptogenesis, estrous cycle disruption, GnRH activity, and circulating hormone levels. This manuscript is largely descriptive, characterizing a model of estrous cycle disruption in a mouse model of temporal lobe epilepsy, but provides limited mechanistic information regarding estrous cycle disruption in epilepsy. This is the first preclinical study to demonstrate direct evidence for changes in GnRH function associated with epilepsy, which is a significant contribution to the field. The study is well done, despite the unavoidable limitations, most of which are well discussed and comments are to further clarify some aspects of the study:

1.One limitation of this study is that it is unclear whether cycle disruption is related to seizure frequency/severity or temporal proximity to a previous seizure event or ictal activity. As it appears that EEG (seizure activity) was not monitored prior to slice collection this should be raised as a possibility; if seizure activity has been monitored this should be reported.

Response: This is an important point. It is correct that we did not do EEG recordings of these mice. At the time these mice were studied, we were not equipped to do the hippocampal EEG recordings needed to quantify seizure burden in this model. As previously stated, we intend to incorporate EEG in our future studies as we agree that quantification of seizure activity would provide valuable information regarding the proximate seizure burden before assessment of, for example, GnRH neuron activity. The following statement in the Discussion has been edited to now read (page 31, line 647): “Whether there is a correlation between hippocampal seizure burden and temporal proximity of recent seizure activity to GnRH neuron functional abnormalities and estrous cycle disruption will be investigated by incorporating electroencephalogram recordings in future studies.”

2. The authors should discuss the relevance of the effect of differences in GnRH activity relative to location (MS and POA vs. AHA).

Response: We have added this point to the Discussion (page 32, line 673): “Furthermore, GnRH neurons form a heterogeneous population with variations, for example, in firing properties (Constantin et al., 2013), neuromodulator receptor expression (Jasoni et al., 2005), and participation in the preovulatory GnRH/LH surge (Hoffman et al., 1993; Wintermantel et al., 2006; Christian and Moenter, 2007) associated with differences in soma location. The relationship between the anatomical location of GnRH neuron somata and heterogeneous neuron properties has not yet been fully characterized, but the present data support a working model in which the location of the GnRH neuron soma helps to shape the functional outcome in the face of epilepsy, in concert with changes in hippocampal-hypothalamic projections differentially targeting the MS, POA, and AHA.”

3. The authors should also discuss what is known regarding the firing patterns required for GnRH function. What is the physiological relevance of these changes in firing patterns? The hormonal measurements are interesting, especially the lower E2 levels is estrus than diestrus, which might be related to the timing of sampling. A discussion on how these hormonal levels compare with other studies in mice would be useful. For the particular study however, it would be worth looking whether there is any correlation between the GnRH firing properties and patterns and the hormones (P4, E2 and/or ratio) for each mouse, regardless of the estrous cycle stage.

Response: The physiological relevance of specific changes in GnRH neuron firing pattern and rate remains an active area of investigation. In terms of a relationship to downstream gonadotropin release, a recent study employing optogenetic activation of GnRH neuron terminals in the median eminence (ME) indicated that light stimulation greater than 10 Hz for at least 2 min is minimally needed to trigger LH pulse secretion (Campos and Herbison, 2014). However, 10 Hz for 2 min is above the levels of GnRH neuron activity observed in most studies. On the other hand, GnRH release from male mouse brain slices was recently demonstrated to be action potential-dependent in the ME, but action potential-independent in POA (Glanowska and Moenter, 2015), indicating complex mechanisms underlying hormone secretion from GnRH neurons. Whether epilepsy drives changes in the relationship between GnRH neuron activity and hormone secretion is yet another unsolved question, but in our opinion it is beyond the scope of the current study, which is only the first to directly demonstrate that there is GnRH neuron dysfunction in an animal model of epilepsy. We agree that a greater understanding of the links between GnRH neuron firing properties and downstream physiology would enhance interpretation of the changes we observed in GnRH neuron firing patterns. However, a discussion of this topic beyond that which we already provided (detailing the potential contributions of changes in burst properties, aberrant tonic spiking on diestrus, and increased quiescence on estrus) would be pure speculation at this time.

Regarding the relationship between GnRH neuron firing pattern and hormone levels, we did not observe direct correlations in relationships between GnRH neuron activity and E2 or P4 levels. Specifically, we tested whether there were correlations for: 1) firing rate -- E2; 2) firing rate -- P4; 3) firing rate -- P4:E2 ratio. No significant correlations were observed (0.26 &lt; p value &lt; 0.89, Pearson correlation). However, it should be noted that the values that we could use in this analysis represented only a small subset of the hormone levels reported in Figure 8, which were calculated from samples combined from mice used for firing activity recordings, current clamp recordings, as well as mice that were not used for in vitro recordings, in order to have a sufficient number of samples for analysis.

The matter of the lower E2 levels measured on estrus compared with diestrus is discussed in response to the following review point.

4. The authors should mention the criteria they use for staging. This is because there is some variance in the way progesterone and estradiol levels in diestrus and estrus are reported across estrous cycle stages in the literature and here. For example, in some studies estradiol is reported higher in estrus than in diestrus and the reverse for progesterone (see Wood et al 2007 in Reproduction), while others agree more with the authors. So it would be appropriate to clarify the criteria and design and perhaps comment on whether such criteria allowed clear differentiation of stages, particularly when cycle was disturbed and where hormonal levels were measured.

Response: Descriptions of the criteria used for identifying the estrous cycle stages are added to the Methods as follows (page 6, line 113): “Smears were classified into each stage based on the following criteria: 1) proestrus: dominated by nucleated epithelial cells; 2) estrus: dominated by cornified epithelial cells; 3) metestrus: both cornified epithelial cells and leukocytes; 4) diestrus I: dominated by leukocytes; 5) diestrus II: few or no cells present.”

We have also specified that these criteria were the same for both control and KA-treated mice (page 6, line 120): “The vaginal smears from KA-injected females did not show major changes in smear cytology characteristics. Therefore, the same criteria were used to classify estrous cycle stages for all mice.”

The E2 ELISA we used has been verified by the University of Virginia Ligand Assay Core to provide the highest accuracy and sensitivity of commercially available mouse/rat estradiol ELISA kits (Haisenleder et al., Endocrinology 2011). In the study by Wood et al. that is mentioned, kits from a different manufacturer were used, and differences in concentrations and the patterns of changes in estradiol levels across estrous cycle stages may be related to the use of different protocols and kits in these experiments. However, we respectfully suggest a discussion of this point is beyond the scope of this manuscript, which (see minor point 6, below) is already quite lengthy.

5. The authors should briefly discuss possible mechanisms underlying the changes in intrinsic excitability and firing patterns of GnRH neurons in KA-treated mice. What underlies the change in AP threshold?

Response: We have added a discussion about potential mechanisms underlying the changes in GnRH neuron AP threshold (page 38, line 801): “Tetrodotoxin-sensitive voltage-gated sodium channels control the action potential depolarization in GnRH neurons (Norberg et al., 2013), and therefore may play important roles in determining action potential threshold. GnRH neuron action potential threshold and latency to firing can also be modulated by E2 feedback, for example through A-type potassium currents (DeFazio and Moenter, 2002). Changes in these and other underlying conductances likely shape the overall shifts in excitability produced both across estrous cycle stages and in response to epilepsy.”

6. The author suggest that the changes in GnRH function may result from the observed changes in estradiol and progesterone levels. The authors should consider experimentally testing this hypothesis, which could contribute more mechanistic information regarding changes in GnRH excitability and cycle disruption in epilepsy.

Response: We fully agree that experimental investigation of the relationship of estradiol/progesterone feedback and the changes in GnRH neuron function would be highly informative. However, full mechanistic dissection of hormone feedback mechanisms is beyond the scope of the current study, which provides the first description of epilepsy-associated changes in GnRH neuron function. We have added this statement to the discussion (page 36, line 762): “Mechanistic investigation of the roles of E2 and P4 feedback in mediating epilepsy-associated changes in GnRH neuron function will be an important aspect of future study, for example through selective hormone replacement in gonadectomized mice, or incorporation of mouse models in which specific steroid hormone receptors are absent or not functional.”

7. The authors should consider discussing how the observed hormone changes relate to the human condition of neuroendocrine disruption in epilepsy patients.

Response: We have added this point to the discussion (page 38, line 784): “Of note, the pattern of changes in E2 and P4 levels detected in this mouse model does not directly match changes in sex steroid levels reported in a limited number of human clinical studies (Herzog et al., 2003; Kalinin and Zheleznova, 2007). Human TLE, however, is a more heterogeneous condition than one animal model can reproduce. The location of seizure origin, the effects of different anti-epileptic drugs, and timing of hormone level measurement in relation to menstrual cycle stage could all profoundly influence the phenotype of changes in sex steroid levels induced by epilepsy. Further exploration of neuroendocrine disruption in both the clinic and in preclinical models is needed to elucidate the spectrum of changes that can be induced by epilepsy and seizure activity, and to determine differential mechanisms that may drive these varying outcomes.”

8. The authors could discuss the implications regarding the evidence of epileptogenesis occurring by 2 weeks whereas the cycle disruption is not evident until 6 weeks.

Response: We have expanded our discussion of this issue, and the text now reads (page 31, line 653): “This time course indicates that the robust pattern of disrupted estrous cyclicity develops gradually after epilepsy is fully established, perhaps reflecting a cumulative effect of seizure burden over time (Raedt et al., 2009). This temporal sequence should provide important insights in future studies about the causal relationships between epileptogenesis, seizure severity, GnRH neuron pathology, and estrous cycle disruption.”

9.The model was reproduced as right hippocampal KA injection. Why was the right side chosen? Was there any pathology on the left hippocampus in any of the mice with estrous cycle abnormalities? In humans it has been proposed that the left TLE is more often associated with hypothalamic / gonadal dysfunction. So that would be an interesting point to discuss for the pathology of the current study and consider for future studies.

Response: The right side was chosen to be consistent with a previous study (Li et al., 2017) that had documented robust disruption of the estrous cycle in mice after targeting KA injection to this side of the hippocampus. Consistent with other studies using the intrahippocampal KA model in mice, gross pathology on the contralateral side was not observed, as shown in the examples in Figure 2. We have added a statement specifying the observed lack of sclerosis and gliosis in the contralateral hippocampus to the Results section (page 15, line 299): “Neither gliosis nor hippocampal sclerosis were observed in the contralateral hippocampus.”

The reviewer is correct that there is evidence in the clinical literature that left vs. right-sided TLE differentially impacts HPG axis function. Of note, as shown in the Li et al. 2017 study, this model of right-sided KA targeting agrees with the clinical findings that right-side TLE individuals do not show cystic ovaries. We agree that systematic comparison of left- vs. right-sided TLE in this mouse model will be an interesting avenue of future studies, but in our opinion this matter is beyond the scope of the present manuscript, which is already quite long.

10. There is no word on hypothalamic pathology and how this correlates with the current results. It would be important for the authors to comment since they must have such data.

Response: We agree that examination of hypothalamic pathology in this model is an important avenue of future investigation. Our purpose in the present studies, however, was to focus on potential changes in GnRH neuron function, as this had not previously been studied in an animal model of epilepsy. We respectfully submit that a comprehensive analysis of hypothalamic pathology, which we intend to perform, is beyond the scope of the current studies. It should be noted, however, that a previous study using the systemic pilocarpine model of TLE in mice did not see major changes in GnRH neuron histology or hypothalamic pathology, despite disrupted estrous cycles. We have added this statement in the discussion (page 33, line 696): “The extent of hypothalamic pathology in the intrahippocampal KA mouse model used here remains unclear. However, neither cell loss nor gliosis were observed in the hypothalamus in a unilateral KA macaque model of epilepsy (Chen et al., 2013), and no GnRH neuron cell loss was observed in a systemic pilocarpine mouse model (Fawley et al., 2012).”

Minor:

1. Please included time of day when sampling was done.

Response: As was stated in the section of the Methods on Estrous Cycle Monitoring, daily vaginal smears were performed between 10 AM to 12 PM. As was stated in the section on Brain Slice Preparation, euthanasia and trunk blood collection was performed between 10-11 AM. As was stated in the section on Targeted Extracellular Recordings, recordings were performed between 11 AM to 3 PM, and current-clamp recordings were performed at the same times. All times are relative to 7 PM lights-off.

2. Significance statement: “PWE are higher risk of reproductive endocrine abnormalities”. Compared to whom? The authors should clarify.

Response: The increased risk is in comparison with the general population. We have edited the Significance Statement to specify this point.

3. Why a 14:10 light/dark cycle was chosen?

Response: This light cycle was chosen to promote cyclicity and enhance breeding in the mouse colony. We have added a statement specifying this point to the Methods (page 5, line 97).

4. The current terms are ‘focal epilepsies’ not partial. Please correct.

Response: We have made this change.

5. Page 14: ‘rarely generate to tonic clonic seizures’: perhaps change to ‘rarely generalize to tonic-clonic seizures’

Response: Thank you for finding this typographical error. We have made this correction.

6.The authors should attempt to make the manuscript more concise. The manuscript is quite long and may be a bit much to tackle for those outside this very specialized field.

Response: Please note that all sections of the manuscript are within the word limits specified by eNeuro. In addition, addressing the major review points requested above required adding multiple points of discussion. Where possible, we have edited the text to reduce and streamline the word count without removing content.

7.The discussion is largely a summary of the results. The discussion could be reworked to discuss the translational relevance of the findings, mechanistic underpinnings, and broader implications.

Response: We have made several edits to the Discussion in order to respond to the major points raised above. We have also made organizational changes and added subheadings to help guide the reader. However, we respectfully disagree that the Discussion was largely a summary of the results. Where needed, results are briefly re-stated in order to provide context for discussion of relevant issues and literature. We have edited the introductory section of the Discussion to streamline this portion.

8.The authors state that the results “indicate that afferent synaptic and/or neuromodulatory inputs likely play a large role in shaping the cycle-stage-dependent shifts in overall firing behavior. While this is true, the intrinsic excitability changes are also interesting and deserve further attention.

Response: We have removed the word ‘large’ to avoid premature overemphasis on the role of afferent inputs.
